# Regulating the regulators: long non-coding RNAs as autophagic controllers in chronic disease management

**DOI:** 10.1186/s12929-024-01092-9

**Published:** 2024-12-23

**Authors:** Aviral Kumar, Kenneth Chun-Hong Yap, Bandari BharathwajChetty, Juncheng Lyu, Mangala Hegde, Mohamed Abbas, Mohammed S. Alqahtani, Soham Khadlikar, Ali Zarrabi, Arezoo Khosravi, Alan Prem Kumar, Ajaikumar B. Kunnumakkara

**Affiliations:** 1 Cancer Biology Laboratory, Department of Biosciences and Bioengineering, Indian Institute of Technology Guwahati (IITG), Guwahati, Assam 781039 India; 2 Department of Pharmacology, Yong Loo Lin School of Medicine, National University of Singapore, Singapore, 117600 Singapore; 3 NUS Center for Cancer Research, Yong Loo Lin School of Medicine, National University of Singapore, Singapore, 119228 Singapore; 4 Electrical Engineering Department, College of Engineering, King Khalid University, 61421 Abha, Saudi Arabia; 5 Radiological Sciences Department, College of Applied Medical Sciences, King Khalid University, 61421 Abha, Saudi Arabia; 6 BioImaging Unit, Space Research Centre, Michael Atiyah Building, University of Leicester, Leicester, LE1 7RH UK; 7 Department of Biomedical Engineering, Faculty of Engineering & Natural Sciences, Istinye University, 34396 Istanbul, Türkiye; 8 Department of Research Analytics, Saveetha Dental College and Hospitals, Saveetha Institute of Medical and Technical Sciences, Saveetha University, Chennai, 600 077 India; 9 Graduate School of Biotechnology and Bioengineering, Yuan Ze University, Taoyuan, 320315 Taiwan; 10 Department of Genetics and Bioengineering, Faculty of Engineering and Natural Sciences, Istanbul Okan University, 34959 Istanbul, Türkiye

**Keywords:** Autophagy, Long non-coding RNAs, Chronic diseases, MicroRNAs, Clinical management

## Abstract

The increasing prevalence of chronic diseases and their associated morbidities demands a deeper understanding of underlying mechanism and causative factors, with the hope of developing novel therapeutic strategies. Autophagy, a conserved biological process, involves the degradation of damaged organelles or protein aggregates to maintain cellular homeostasis. Disruption of this crucial process leads to increased genomic instability, accumulation of reactive oxygen species (ROS), decreased mitochondrial functions, and suppression of ubiquitination, leading to overall decline in quality of intracellular components. Such deregulation has been implicated in a wide range of pathological conditions such as cancer, cardiovascular, inflammatory, and neurological disorders. This review explores the role of long non-coding RNAs (lncRNAs) as modulators of transcriptional and post-transcriptional gene expression, regulating diverse physiological process like proliferation, development, immunity, and metabolism. Moreover, lncRNAs are known to sequester autophagy related microRNAs by functioning as competing endogenous RNAs (ceRNAs), thereby regulating this vital process. In the present review, we delineate the multitiered regulation of lncRNAs in the autophagic dysfunction of various pathological diseases. Moreover, by highlighting recent findings on the modulation of lncRNAs in different stages of autophagy, and the emerging clinical landscape that recognizes lncRNAs in disease diagnosis and therapy, this review highlights the potential of lncRNAs as biomarkers and therapeutic targets in clinical settings of different stages of autophagic process by regulating ATG and its target genes. This focus on lncRNAs could lead to breakthroughs in personalized medicine, offering new avenues for diagnosis and treatment of complex diseases.

**Graphical Abstract:**

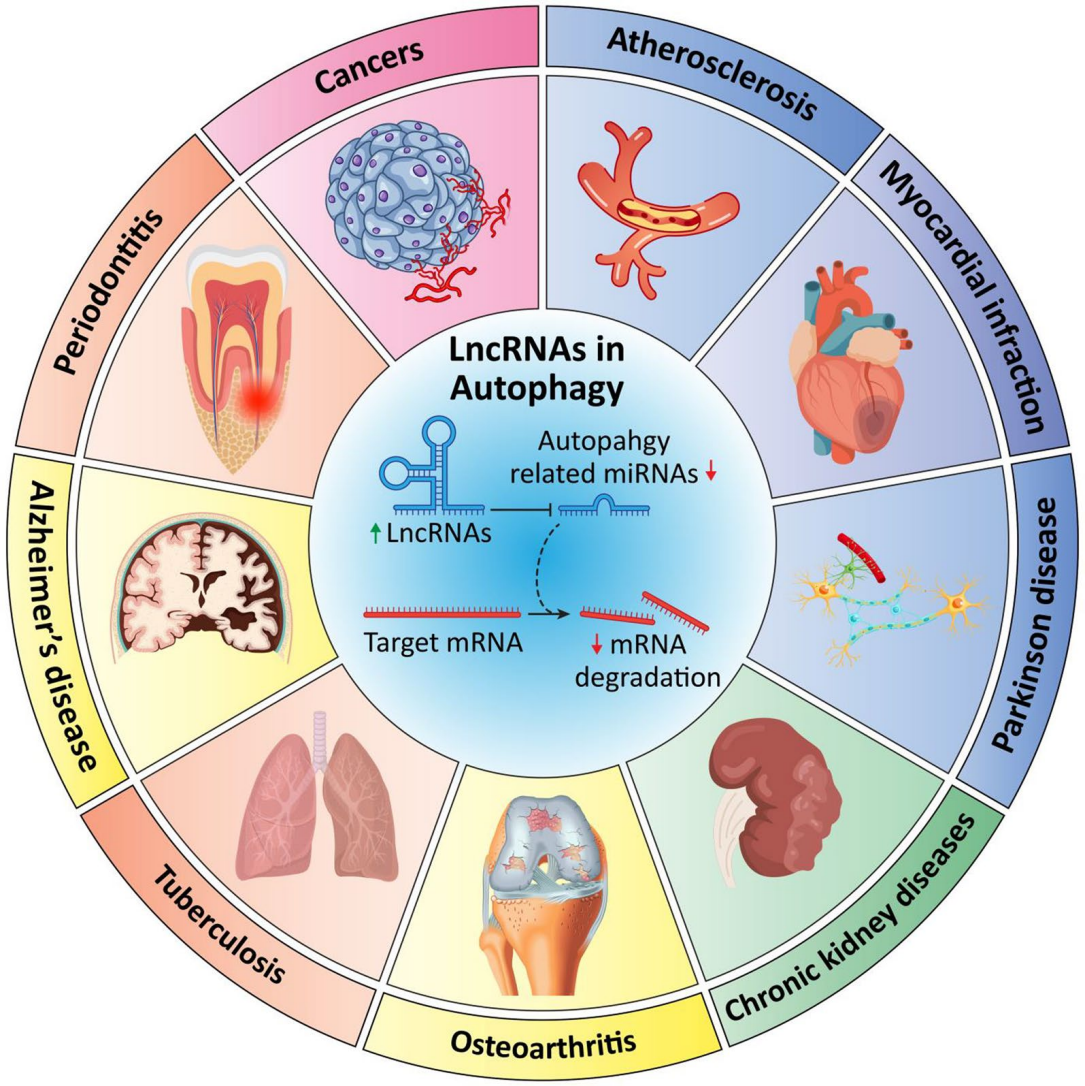

## Introduction

Chronic diseases have become the leading challenge of the healthcare sector with increasing mortality and morbidity worldwide. The treatment of chronic diseases poses unique challenges due to their long-term nature, complex etiology, and varying disease progression among individuals [1–5]. The management of chronic diseases requires a comprehensive and multifaceted approach to address the diverse needs of patients [1, 6–11]. Hence, it is crucial to understand the causative factors underlying these multifactorial diseases to develop safe, and efficacious treatment strategies.

**Fig. 1:**
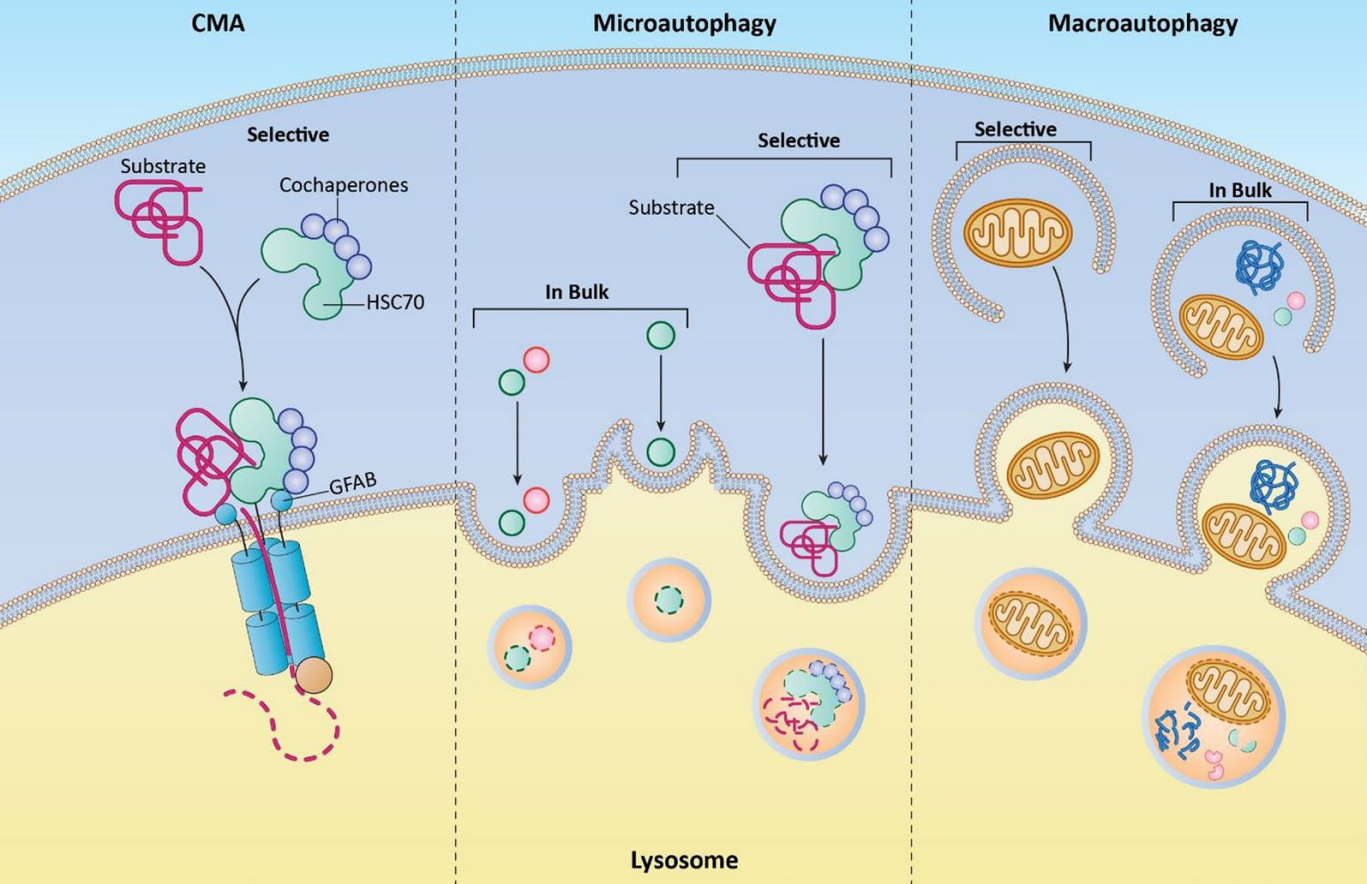
Selective/non-selective uptake of proteins or organelles into the lysosome for degradation through CMA, Microautophagy, and Macroautophagy. This figure illustrates the mechanisms by which proteins and organelles are selectively or non-selectively transported to the lysosome for degradation. CMA involves the selective uptake of soluble cytosolic proteins into the lysosome, facilitated by specific chaperones that recognize and bind to target proteins containing a KFERQ-like motif. Microautophagy refers to the direct engulfment of small portions of cytoplasm or organelles by the lysosomal membrane, leading to the internalization and degradation of the cargo. Macroautophagy, involves the formation of a double-membraned vesicle called the autophagosome, which engulfs larger cellular components before fusing with the lysosome for degradation

Autophagy, a fundamental biological process is involved in maintenance of cellular homeostasis by recycling impaired organelles, aggregates of proteins, and intracellular pathogens [12]. It is a dynamic, regulated cellular process that involves the degradation of cellular components within specialized compartments called autophagosomes [13]. The word “autophagy” originates from the Greek texts “auto” (self) and “phagy” (eating), relating the phenomenon of digestion of impaired components by the cells [14]. With the increase in the understanding of the autophagic process and the underlying mechanisms involved in it, autophagy have been stratified into different types such as macroautophagy, microautophagy, and chaperone-mediated autophagy (CMA) based on the cargo content and the biogenesis mechanism. Macroautophagy, often referred to as autophagy, has been the most significantly studied type where the formation of autophagosomes take place that engulf damaged organelles, proteins, and other cytoplasmic material. Further, fusion of autophagosomes with lysosomes forms the autolysosomes, where the enclosed contents are processed by lysosomal enzymes, releasing basic building blocks that can be reused by the cell [15, 16]. Microautophagy is a distinct form of autophagy characterized by the direct engulfment of cytoplasmic material by invagination or protrusion of the lysosomal membrane. This process allows for the selective or non-selective uptake of proteins or organelles directly into the lysosome for degradation (Fig. 1) [17]. Microautophagy is considered a constitutive process that occurs continuously and contributes to the turnover of cellular components. Although less understood compared to macroautophagy, microautophagy is emerging as an important mechanism for protein quality control and organelle homeostasis [13, 18]. On the other hand, CMA is a selective form of autophagy that targets and degrades specific proteins (Fig. 1). In this process of CMA, it recognizes specific amino acid motifs in target proteins by chaperones present in the cytosol. Then, chaperones along with the target proteins are delivered to the lysosome for degradation [19].

Accumulating evidence implicates the deregulation of autophagic process in various chronic diseases, including cancer, neurodegenerative disorders, cardiovascular diseases, and inflammatory conditions [20]. Autophagy dysfunction has been linked in the initiation and progression of various neurodegenerative disorders such as Alzheimer's, Parkinson's, and Huntington's disease. There has been reports where build-up of misfolded proteins and impaired clearance mechanisms are responsible for the formation of protein aggregates, contributing to neuronal toxicity and cell death [21, 22]. Enhancing autophagy has shown therapeutic potential in mitigating neurodegenerative pathology in various experimental models. In cancer, autophagy has a dual role, acting both as an oncogenic and tumor suppressor mechanism. In the initial stages of tumorigenesis, autophagy prevents the accumulation of damaged components and genomic instability, acting as a tumor suppressor [23–25]. However, in established tumors, autophagy enables cancer cell survival under nutrient-deprived conditions and promotes resistance to therapy [26].

Long non-coding RNAs (lncRNAs) are a varied class of non-coding RNA molecules with the absence of protein-coding elements but play important roles in gene regulation and cellular processes [27, 28]. Although initially considered as “transcriptional noise,” recent research has unraveled their vital functions in diverse biological processes, such as regulation of gene expression, chromatin remodeling, and post-transcriptional modifications. In recent years, emerging evidence has suggested that lncRNAs have a substantial impact on autophagy, a cellular process essential for maintaining cellular homeostasis and the clearance of damaged organelles and proteins [29]. Several lncRNAs have been identified as direct regulators of autophagy [30]. For example, the lncRNA MEG3 promoted autophagy by interacting with the autophagy-related gene 5 (ATG5) and facilitating the formation of autophagosomes [31]. Conversely, lncRNA BACE1-AS inhibit autophagy by sequestering miRNAs and preventing the degradation of protein involved in amyloid-beta production and Alzheimer's disease pathogenesis [32]. Some lncRNAs exert their influence on transcriptional activity of autophagy by modulating the expression of key genes related to autophagy [33–38]. The lncRNA HOTAIR promoted autophagy by binding to the transcription factor E2F1 and facilitating its recruitment to the promoter areas of autophagy-related genes. This interaction enhances autophagy induction and contributes to cellular homeostasis [39]. This review explores the concept of lncRNAs modulating autophagy and its significance in the pathophysiology of chronic diseases.

## LncRNAs as regulators of autophagic process

Autophagy is an intricate, evolutionary conserved and agile process eliminating misfolded proteins, damaged or aged organelles and mutated proteins, that contains three types such as microautophagy, macroautophagy and chaperone-mediated autophagy, where the macroautophagy has received significant attention and thorough investigation [40]. Here we have discussed the prominence of various lncRNAs regulating the different stages of autophagy by modulating the ATG and its downstream target genes (Table 1).

**Table 1:** Autophagy modulating lncRNAs and their effect in different chronic diseases

	LncRNA	Target miRNA/gene	Mechanism of action on autophagy	Effect after overexpression/knockdown	Ref
*Cancers*					
*Acute Myeloid Leukemia*	LINC00265 ^b^	miR-485-5p	↓LC3-II/LC3-I ratio, ↓Beclin-1, ↑p62	↓IRF2, ↑Apoptosis	[41]
	UCA1 ^a^	miR-96-5p	↑ATG7, ↑Beclin-1	↑Proliferation	[42]
	DANCR ^a^	miR-874-3p	↑ATG16L1, ↑LC3-II, ↓SQSTM1/p62	↑Cytarabine resistance	[43]
*Breast cancer*	DANCR ^b^	miR-758-3p	↑ATG5, ↑LC3B, ↓Beclin-1	↑Caspase 3, ↑Caspase 9, ↑Bax, ↓Bcl-2	[44]
	GAS5 ^a^	–	↑LC3B, ↑Beclin-1, ↑ULK1, ↑ULK2	↑Chemosensitivity	[45]
	H19 ^b^	–	↓Beclin-1, ↓LC3-II	↓Tamoxifen resistance, ↑DNMT3B	[46]
	OTUD6B-AS1 ^a^	miR-26a-5p	↑LC3B-II	↑γ-H2AX, ↓p-ATR, ↓p-ATM, ↓p-RAD51	[47]
*Bladder cancer*	MEG3 ^b^	–	↑LC3-II	↓Apoptosis, ↓G0/G1 phase populations	[48]
	ADAMTS9-AS1 ^b^	AMDAMT9	↑Beclin-1, ↑LC3-II/LC3-I ratio	↑Caspase 9, ↑Bax, ↓Vimentin, ↓N-cadherin, ↓Snail, ↑E-cadherin, ↓p62, ↓Bcl-2, ↓PIK3CB, ↓p-AKT, ↓p-mTOR	[49]
*Cervical cancer*	ROR1-AS1 ^b^	miR-670-3p	↓Beclin 1, ↑LC3-I, ↓LC3-II	↓Proliferation, ↑Apoptosis	[50]
	RP11-381N20.2 ^a^	–	↓Paclitaxel-induced autophagy, ↓ATG7	↑Chemosensitivity	[51]
	MLLT4-AS1^a^	Myosin-9	↑LC3-II, ↓p62	↓Migration, ↓Invasion,	[52]
*Clear cell renal cell carcinoma*	TUG1 ^b^	miR-31-5p	↑LC3-II/LC3-I ratio, ↓p62	↓PCNA, ↑cle-Caspase 3, ↓FLOT1	[53]
*Colon cancer*	EGOT ^a^	–	↓Beclin-1, ↑p62, ↓LC3-II/LC3-I	↓cle-Caspase 3, ↓Bax, ↑Bcl-2, ↑Proliferation, ↑Invasion	[54]
	CASC2 ^a^	miR-214	↑Beclin-1, ↑LC3-II	↓TRIM16, ↑Bax, ↓Bcl-2, ↑cle-Caspase 3, ↓Proliferation	[55]
	LINC00858 ^b^	–	↑Beclin-1, ↑LC3II/I	↑Bax, ↓Bcl-2, ↑cle-Caspase 3, ↑p27	[56]
	KCNQ1OT1 ^b^	miR-34a	↓Atg4B, ↓LC3II,	↑cle-PARP, ↑Chemosensitivity, ↓Proliferation	[37]
*Colorectal cancer*	NEAT1 ^b^	miR-34a-5p	↓ATG9A, ↓ATG4B, ↓Beclin-1, ↓LC3II/I ratio, ↓ULK1	↑cle-Caspase 3, ↓HMGB1, ↑Chemosensitivity	[57]
	SLCO4A1-AS1 ^a^	miR-508-3p	↑LC3B-II	↑Proliferation, ↓Apoptosis	[58]
	SNHG14 ^b^	miR-186	↓ATG14, ↓LC3B	↓Proliferation, ↓Migration, ↓Invasion, ↓Cisplatin resistance	[34]
	UCA1 ^b^	miR-23b-3p	↓LC3-II/LC3-I ratio, ↓Beclin-1, ↑p62	↑Bax, ↑Caspase 3, ↓5-FU resistance, ↓ZNF281	[59]
	MALAT1 ^b^	miR-101	↓LC3-II/LC3-I ratio, ↑p62	↓Proliferation, ↑cle-Caspase 3	[60]
	CPS1-IT1 ^a^	–	↓LC3-II, ↓Beclin-1,	↓HIF-1α, ↓N-cadherin, ↓Vimentin, ↑E-cadherin, ↑ZO-1	[61]
	H19 ^a^	miR-194-5p	↑LC3-II, ↓p62	↑Proliferation, ↑SIRT1, ↑Chemoresistance	[62]
	SNHG6 ^b^	miR-26a-5p	↓p-ULK1, ↓ATG13, ↓ULK1	↓Proliferation, ↑cle-Caspase 3, ↑cle- PARP, ↓Chemoresistance	[63]
	CASC9 ^b^	–	↑LC3B-II, ↓p62	↓Proliferation, ↓Migration, ↓Vimentin, ↑E-cadherin, ↑p-AMPKα/AMPKα, ↓p-AKT, ↓p-mTOR	[64]
	SNHG8 ^a^	miR-588	↑LC3-II, ↑ATG7,	↑Proliferation	[65]
	TUG1 ^a^	miR-195-5p	↑LC3II, ↑Beclin-1	↑Proliferation, ↓p53, ↓Bax, ↑Bcl-2, ↓Caspase 3, ↑HDGF, ↑DDX5, ↑β-catenin	[66]
*Gastric cancer*	SNHG11 ^b^	miR-483-3p/miR-1276	↓LC3-II/LC3-I ratio, ↑p62, ↓LAMP1	↓Twist, ↓Nanog, ↓LRG5, ↓CD133, ↓EpCAM, ↓Sox2, ↓Bcl-2, ↑Bax, ↓MMP-2, ↓MMP-7, ↑E-cadherin, ↓N-cadherin, ↑GSK-3β, ↓β-catenin, ↑cle- PARP, ↑cle-Caspase 3, ↑cle-Caspase 6	[67]
	JPX ^b^	miR-197	–	↓Proliferation, ↓Migration, ↓Invasion	[68]
	LINC01572 ^b^	miR-497-5p	↓Autophagy	↓Proliferation, ↓Migration, ↓Invasion, ↓Cisplatin resistance	[69]
	CRNDE ^a^	–	↓LC3-II	↑Apoptosis, ↑cle- PARP, ↑cle-Caspase 3, ↓Chemoresistance	[70]
	MALAT1 ^a^	miR-204	↑LC3B	↑Proliferation, ↑Ki67, ↑TRMP3	[71]
	MALAT1 ^b^	miR-23b-3p	↓LC3-II/LC3-I ratio, ↑p62, ↓ATG12	↓Chemoresistance	[72]
	MALAT1 ^a^	miR-30b	↑LC3-II, ↓p62, ↑ATG5	↑Proliferation, ↑Cisplatin resistance	[73]
	HULC ^a^	–	↑LC3-II/LC3-I, ↑Beclin-1, ↓p62	↑FoxM1, ↑MDR1, ↑Cisplatin resistance	[74]
	HAGLROS ^b^	miR-100-5p	↑LC3-II/LC3-I, ↓p62	↓p-mTOR, ↓mTOR, ↓p-4E-BP1, ↓Proliferation, ↓Migration, ↓Invasion	[75]
	EIF3J-DT ^b^	miR-188-3p	↓LC3-II, ↓ATG14	↓Proliferation, ↑cle-PARP, ↑cle-Caspase 3, ↓Chemoresistance	[76]
	DANCR ^b^	miR-194	↑LC3-II/LC3-I ratio, ↑Beclin-1	↑Apoptosis	[77]
	CCAT1 ^a^	miR-140-3p	↑LC3A/B, ↑Beclin-1, ↑ATG5, ↑ATG12	↑Proliferation, ↑Migration, ↑Invasion	[78]
	LIT3527 ^b^	–	↑LC3-II	↓Proliferation, ↑Apoptosis, ↓Migration, ↓p-AKT, ↓p-mTOR, ↓p-ERK, ↓4EBP1, ↓Metastasis	[79]
	FEZF1-AS1 ^b^	–	↓LC3-II, ↓ATG5	↑Bax, ↓Bcl-2, ↑cle-Caspase 3, ↓MDR1, ↓MPR1, ↓S-phase cell populations, ↓Chemoresistance	[33]
	LINC00963 ^b^	miR-4458	↓LC3-II, ↑p62	↓Proliferation, ↓Migration	[35]
*Glioblastoma*	LINC00470 ^a^	miR-101	↓LC3-II, ↓ATG7, ↓ATG3, ↓Beclin-1	↑ELFN2, ↓Dicer	[38]
*Glioma*	MALAT1 ^b^	miR-101-3p	↓LC3-II, ↑p62, ↓ATG4D	↓Proliferation, ↓STMN1, ↓RAB5A	[80]
	CASC2 ^a^	miR-193a-5p	↓LC3-II, ↓Beclin-1, ↑p62	↑mTOR, ↓Migration	[81]
	GAS5 ^a^	–	↓LC3-II, ↑p62	↓Proliferation, ↑p-mTOR ↑Chemosenstivity	[82]
	AC023115.3 ^b^	miR-26a	↑LC3-II, ↓p62	↓cle-Caspase 3, ↓cle- PARP	[83]
	Linc-RA1 ^a^	–	↓LC3B-II/I ratio, ↑p62	↓% DNA damage, ↓% Irradiation-induced death, ↑H2Bub1, ↓γ-H2AX, ↑Radioresistance	[84]
	H19 ^a^	–	↓Autophagy, ↑p-ULK1	↑Proliferation, ↑Migration, ↓p-mTOR	[85]
	LINC00470 ^a^	miR-580-3p	↓LC3-II/LC3-I, ↓Beclin-1, ↑p62	↑Proliferation, ↓G1phase cell population, ↑p-PI3K, ↑p-mTOR, ↑p-AKT	[86]
	Lnc-NLC1-C ^b^	–	↓LC3II/I, ↓p62, ↑ATG9	↓Proliferation, ↓Migration, ↓Invasion, ↑ROS generation, ↑Rab1, ↓PRDX-3	[87]
	DRAIC ^a^	–	↓LC3-II, ↓p62, ↓p-ULK1 (S757)	↓Migration, ↓Invasion, ↓p-S6K, ↑p-AMPK, ↑p-RPTOR, ↑p-FOXO3a	[88]
*Head and neck squamous cell carcinoma*	LINC00460 ^b^	miR-206	↑LC3-II/I ratio, ↑Beclin-1	↓STC2, ↓AKT, ↓ERK, ↓p-ERK, ↓p-AKT, ↑G0/G1-phase cell arrest, ↑Bax, ↑cle-PARP, ↑cle-Caspase 3	[89]
	EIF3J-DT ^b^	–	↓LC3-II, ↑p62, ↓ATG14	↓Proliferation, ↓Colony formation, ↑Apoptosis, ↑cle-Caspase 3, ↑cle- PARP, ↓Cyclin D1, ↑p21, ↑Taxol sensitivity	[90]
*Hepatocellular cancer*	SNHG11 ^b^	mir-184	↓AGO2, ↓Beclin-1, ↓LC3-II/I ratio	↑cle-Caspase 3, ↓Migration, ↓Invasion	[91]
	HOTAIR ^a^	–	↑LC3-II, ↑ATG3, ↑ATG7	↑Proliferation	[92]
	H19 ^b^	–	↓LC3-II/1 ratio, ↓Beclin-1, ↑p62	↑Proliferation, ↑G0/G1-phase cell population, ↓cle-Caspase 3, ↓cle-Caspase 9, ↑Bcl-2, ↓Cyt c, ↑p-PI3K, ↑p-AKT, ↑p-mTOR	[93]
	PVT1 ^a^	miR-365	↑LC3-II, ↑ATG3	↑Proliferation, ↑Ki67	[94]
	CCAT1 ^a^	miR-181a-5p	↑LC3-II, ↓p62, ↑ATG7	↑Proliferation	[95]
	MEG3 ^a^	–	↓LC3-II/LC3-I, ↓Beclin-1	↓Proliferation, ↓ILF3, ↑p-PI3K, ↑p-AKT, ↑p-mTOR	[96]
	HNF1A-AS1 ^a^	miR-30b-5p	↑LC3BII/I, ↓p62, ↑ATG5,	↑Proliferation, ↑Bcl-2	[97]
	MCM3AP-AS1 ^b^	miR-455	-	↓Migration, ↓Vessel formation	[98]
	NBR2 ^a^	–	↓LC3 II/I ratio, ↓Beclin-1, ↑p62,	↓Proliferation, ↓Migration, ↓Invasion, ↓p-ERK, ↓p-JNK	[99]
	NEAT1 ^a^	miR-204	↑LC3-II/I ratio, ↑ATG3	↓Sorafenib-induced growth inhibition, ↑p-AKT, ↑p-mTOR,	[36]
	DCST1-AS1 ^b^	–	↑Autophagy	↓Proliferation, ↓Migration, ↑Apoptosis	[100]
	HAGLROS ^b^	miR-5095	↓LC3 II/I ratio, ↓Beclin-1, ↑p62	↑Bax, ↑cle-Caspase 3, ↑cle-Caspase 9, ↓Bcl-2, ↓p-PI3K, ↓p-AKT, ↓p-mTOR, ↑PTEN	[101]
	DANCR ^b^	miR-222-3p	↓Autophagy	↓Proliferation	[102]
	HULC ^a^	miR-15a	↑LC3 II/I ratio	↑Proliferation, ↑Sirt1, ↓PTEN, ↑JAK, ↑PKM2, ↑CDK2, ↑p-PI3K, ↑p-AKT, ↑p-mTOR, ↑Jun, ↑Survivin	[103]
	ATB ^a^	–	↑LC3 II/I ratio, ↑ATG5	↑Proliferation, ↓p-YAP	[104]
	CRNDE ^a^	miR-543	↑ATG4B, ↑LC3-II/I ratio, ↓p62	–	[105]
	RP11-295G20.2 ^a^	PTEN	↓LC3B	↓PTEN, ↑p-AKT, ↓FOXO3a	[106]
	CCAT2 ^b^	miR-4496/ELAVL1	↓LC3 II/I ratio, ↓Beclin-1, ↑p62	↓Migration, ↓Invasion	[107]
	HnRNPU-AS1 ^a^	miR-556-3p/miR-580-3p	↑Autophagy	↓Proliferation, ↓Migration,	[108]
*Hypoxic tumor*	LincRNA-p21 ^b^	–	↓LC3 II, ↑p62	↓Proliferation, ↑G2/M arrest of cell populations, ↓Migration, ↓HIF-1α,	[109]
*Laryngeal squamous cell carcinoma*	H19 ^b^	miR-107	↓LC3 II/I ratio, ↓Beclin-1, ↑p62, ↓LAMP2	↓Chemoresistance	[110]
*Lung cancer*	MSTO2P ^b^	–	↓Agt5, ↓LC-3II	↓Proliferation, ↓EZH2	[111]
	LCPAT1 ^b^	RCC2	Autophagy halted after CSE/ PM2.5 exposure	↓Proliferation, ↓Migration, ↓Invasion	[112]
	LINC00857 ^b^	YBX1	↑LC3 II/I ratio	↓Proliferation, ↑cle-PARP, ↓YBX1, ↓p-MET, ↑p-AMPKa	[113]
	PANDAR ^a^	–	↑Autophagy, ↑Beclin-1	↓Proliferation	[114]
	MITA1 ^a^	–	↑LC3 II/I ratio, ↑Beclin-1, ↓p62	↓Apoptosis, ↑Gefitinib resistance	[115]
	LINC01279 ^b^	SIN3A	↑Beclin-1, ↓p62	↓Proliferation, ↓Migration, ↓Invasion, ↑Apoptosis, ↓p-ERK, ↓FAK, ↑p53, ↓p21,	[116]
	LINC00265 ^b^	SIN3A	↑LC3 II/I ratio, ↑Beclin-1, ↓p62	↓Proliferation, ↓Migration, ↓Invasion, ↑Apoptosis, ↓p-mTOR, ↓p-P70, ↑p-AMPK	[117]
*Lymphoma*	BCYRN1 ^a^	–	↑Autophagy, ↑Beclin-1, ↑LC3-II	↑Proliferation, ↑Bcl-2, ↑Cyclin D1, ↓p53, ↓Bax, ↓p21, ↓p-mTOR, ↓p-AKT	[118]
* Multiple myeloma*	MALAT1 ^b^	HMGB1	↓Beclin-1, ↓LC3B	↓Proliferation, ↑Apoptosis, ↓HMBG1	[119]
*Nasopharyngeal cancer*	MEG3 ^a^	miR-21	↑LC3-II/I ratio, ↑Beclin-1, ↓p62	↑Bax, ↑cle-Caspase 3, ↓Bcl-2, ↑PTEN	[120]
	LINC00313 ^b^	–	↑LC3-II, ↓p62	↓Proliferation, ↓SOX2, ↓Oct4, ↓Nanog, ↓CD133, ↓PTBP1, ↓STIM1, ↓p-AKT, ↓p-mTOR, ↓p-P70S6K	[121]
*Neuroblastoma*	SNHG7 ^b^	miR-329-3p	↓LC3B-II/LC3B-I, ↓Beclin-1, ↑p62	↓Proliferation, ↓Chemoresistance	[122]
*Non-small cell lung cancer*	UCA1 ^b^	miR-185-5p	↓LC3-II/I ratio, ↓Beclin-1, ↑p62,	↓Proliferation, ↓Ki67, ↑Caspase 3, ↓WISP2, ↓β-catenin, ↓TCF4	[123]
	NBAT1 ^b^	PSMD10	↑LC3-II, ↓p62, ↑ATG7	↑PSMD10, ↑Proliferation, ↑Chemoresistance	[124]
	BLACAT1 ^a^	miR-17	↑LC3-II/I ratio, ↑Beclin-1, ↑ATG7	↑Chemoresistance, ↑Proliferation, ↑MRP1	[125]
	GAS5 ^a^	–	↓LC3-II	↓Chemoresistance, ↓Proliferation	[126]
	PVT1 ^b^	miR-216b	↓LC3B-II/I, ↑p62, ↓Beclin-1	↑Apoptosis, ↑Cisplatin sensitivity,	[127]
*Osteosarcoma*	CTA ^a^	miR-210	↓LC3-II/LC3-I	↓BNIP3/BNIP3L, ↑cle-Caspase 3, ↑Doxorubicin sensitivity, ↑Apoptosis	[128]
	SNHG15 ^b^	miR-141	↓LC3-II/LC3-I, ↓ATG5, ↑p62	↓Proliferation, ↓Migration, ↓Invasion	[129]
	SNHG6 ^b^	miR-26a-5p	↓ULK1	↑ATF3, ↑cle-Caspase 3, ↓Proliferation, ↓Migration, ↓Invasion	[130]
*Ovarian cancer*	HOXA11-AS ^b^	–	↑LC3-II/I ratio, ↑Beclin-1, ↓p62	↓Migration, ↓Invasion, ↑Cisplatin sensitivity	[131]
	TUG1 ^b^	miR-29b-3p	↓Beclin-1, ↓LC3B-II/I	↑cle-Caspase 3, ↑cle-Caspase 7, ↓Proliferation, ↑Paclitaxel sensitivity	[132]
	XIST ^b^	miR-506-3p	↓LC3-II/I ratio, ↑p62	↑Bax, ↓Bcl-2, ↑Carboplatin sensitivity	[133]
*Pancreatic cancer*	LINC01207 ^b^	miR-143-5p	↑LC3-II, ↑Beclin-1, ↓p62	↓AGR2, ↓Cell growth, ↑Apoptosis, ↓Bcl-2/Bax	[134]
	PVT1 ^b^	miR-619-5p	↓ATG14, ↓LC3-II, ↑p62	↓Pygo2, ↓Cyclin-D1, ↓c-Myc, ↓Axin2, ↓Gemcitabine resistance	[135]
	MALAT1 ^b^	HuR	↓LC3B II/I, ↑p62, ↓LAMP-2	↓MMP-3, ↓MUC4	[136]
	SNHG14 ^b^	miR-101	↓ATG4D	↓RAB5A, ↓Gemcitabine resistance, ↓Migration, ↓Invasion	[137]
	ANRIL ^b^	miR-181a	↓LC3-II, ↑Beclin-1	↓HMGB1, ↓Proliferation, ↓Snail, ↓Vimentin, ↑E- cadherin, ↓N-cadherin	[138]
*Papillary thyroid cancer*	RP11-476D10.1 ^b^	miR-138-5p	↑Beclin-1, ↑LC3B	↓LRRK2, ↑Bax ↓Bcl-2	[139]
	BANCR ^b^	–	↓LC3-II/LC3-I	↑Apoptosis, ↑Cell population in the G1 phase	[140]
*Prostate cancer*	HULC ^b^	–	↑p-Beclin-1, ↑LC3B-II	↑Bax, ↑Caspase 3, ↓PCNA, ↓Cyclin D1, ↑Irradiation sensitivity	[141]
	SNHG1 ^b^	EZH2	↑LC3-II, ↑Beclin-1, ↓p62	↓p-PI3K, ↓p-AKT, ↓p-mTOR, ↓p-p70S6K, ↓Wnt1, ↓β-catenin, ↓c-Myc, ↓Cyclin D1, ↓EZH2	[142]
	PRRT3-AS1 ^b^	PPARγ	↑LC3-A, ↑LC3B, ↑Beclin-1	↓p-S6K1, ↓NF-κB1, ↓COX2, ↓p-4EPB1, ↓PCNA, ↓Ki67, ↑PPARγ, ↑Bax, ↑cle-Caspase 3, ↓Bcl-2, ↓Migration, ↓Invasion	[143]
*Renal cell carcinoma*	LBX2-AS1 ^b^	–	↑LC3-II, ↑NIX/BNIP3L	↓Proliferation, ↓Migration, ↓FOXO3A	[144]
	SNHG1 ^b^	PTBP1	↓LC3-II, ↓Beclin-1, ↑p62, ↓ATG7	↓Proliferation, ↓Migration, ↓Invasion, ↑Apoptosis, ↓Sunitinib resistance	[145]
*Retinoblastoma*	MALAT1 ^b^	miR-124	↓LC3-II, ↓Beclin-1, ↑p62	–	[146]
*Uveal melanoma*	ZNNT1 ^a^	–	↑ATG12, ↓p62	↓Tumor cell growth, ↓Migration, ↓Invasion	[147]
Cardiovascular diseases					
*Atherosclerosis*	FA2H-2^b^	MLKL	↓LC3-II, ↓LAMP-1, ↑p62	↑IL-6, ↑VCAM-1, ↑MCP-1, ↑IL-8, ↑IL-18, ↑IL-1β, ↑TNF-α, ↓IL-10, ↑p-PI3K, ↑p-AKT, ↑p-mTOR, ↓p-AMPK	[148]
	MALAT1^b^	miR-15b-5p	↑LC3-II, ↑ATG1,	↓p-mTOR, ↓p-ERK1/2, ↓VCAM-1, ↓ICAM-1	[149]
	LOC107986345^a^	miR-128-3p	↑MAP1LC3B2, ↓p62	↑EPHB2, ↓ICAM-1	[150]
	MALAT1^a^	miR-216a-5p	↑Beclin-1, ↑LC3 II/I	↓Apoptosis, ↓Caspase 3	[151]
	GAS5^b^	miR-26a	↑LC3 II/I, ↓p62	↓Apoptosis	[152]
	ZNF295-AS1^b^	miR-508-5p	↓LC3B, ↓ATG7	↑Proliferation	[153]
	CTBP1-AS2^a^	miR-195-5p	↑LC3, ↑Beclin-1, ↑ATG14	↓Proliferation, ↓Colony formation, ↓PCNA, ↓Ki67	[154]
	TUG1^b^	–	↑LC3 II/I, ↓p62, ↑ATG3	↓Proliferation, ↓Migration, ↑p-AMPK/AMPK, ↓p-mTOR/mTOR	[155]
	GAS5 ^b^	miR-193-5p	↑LC3 II/I, ↓p62	↓SRSF10	[156]
*Congenital heart disease*	NEAT1^a^	miR-181b	↓LC3 II/I, ↓Beclin-1, ↑p62	↑Proliferation, ↓Apoptosis, ↓p53, ↑Bcl-2, ↓Bax, ↓cle-caspase 3, ↑p/t-PI3K, ↑p/t-AKT, ↑p/t-mTOR, ↑p/t-STAT3	[157]
*Heart failure*	MEG3 ^b^	–	↓Beclin-1, ↓LC3 II/I, ↑p62	↑Cardiac function, ↓NPPA, ↓NPPB, ↓MYH7, ↑Bcl-2/Bax ratio, ↑p-AKT, ↑p-GSK3β	[158]
*Myocardial infraction*	MALAT1^b^	miR-558	↓ULK1, ↓LC3‐II/I	↑cle-PARP, ↓Proliferation, ↑Apoptosis	[159]
	MALAT1^a^	-	↓LC3 II/I, ↓Beclin-1	↓Proliferation, ↑Apoptosis ↓TSC2, ↑H3K27me3, ↑p-mTOR, ↑Caspase 3, ↑Bax, ↓Bcl-2	[160]
	MALAT1^b^	miR-30a	↑Beclin-1	–	[161]
	MALAT1^a^	–	↓Autophagy	↑Apoptosis	[162]
	MALAT1^a^	miR-206	↑ATG3	↑CK‐MB, ↑LDH	[163]
	MALAT1^b^	miR-4465	↓ULK1, ↓LC3-II/I, ↑p62	Hypoxia-induced cell injury	[164]
	XIST^b^	miR-133a	↓LC3 II/I, ↓Beclin-1	↑Proliferation, ↓Apoptosis, ↓Myocardial I/R injury	[165]
	DANCR^a^	miR-6324	↑Beclin-1, ↑LC3 II/I, ↓p62	↑Proliferation, ↓Apoptosis, ↑Bcl-2, ↓Bax, ↓cle-caspase 3, ↓cle-caspase 9, ↑p-IRE1α,↑Xbp1s	[166]
	NEAT1^b, c^	miR-378a-3p	↓ATG12, ↓LC3, ↑p62	↑Proliferation, ↓Apoptosis, Migration	[167]
	MHRT^a, c^	–	↑Beclin-1, ↑LC3 II/I	↓Apoptosis, ↑LVEF, ↑LVFS, ↓Myocardial fibrosis, ↓Bax/Bcl-2 ratio, ↓cle-caspase 3	[168]
	TUG1^b^	miR-142-3p	↓Beclin-1, ↓LC3 II/I, ↑p62	↓I/R-induced infarction size, ↓Apoptosis	[169]
	KCNQ1OT1^b^	miR-26a-5p	↓Beclin-1, ↓LC3 II/I, ↓ATG12	↓cle-Caspase 3, ↑Bcl-2, ↓Bax	[170]
	AK088388^b^	miR-30a	↓Beclin-1, ↓LC3 II/I	↑Proliferation, ↓Apoptosis	[171]
	PVT1^b^	miR-186	↓Beclin-1, ↓LC3 II/I, ↑p62	↑Proliferation, ↓Apoptosis, ↑Bcl-2, ↓Bax, ↓cle-caspase 3	[172]
	NEAT1^a^	-	↑LC3 II/I, ↓p62, ↑ATG5, ↑ATG7	↑LDH, ↓SOD, ↑Foxo1↑CK-MB, ↑LVEDP, ↑I/R injuries	[173]
	APF^b^	miR-188-3p	↓LC3 II/I, ↓autophagic vesicles, ↓ATG7	↑Myocardial function, ↓Cell death	[174]
	H19^a^	–	↑Beclin-1, ↑LC3 II/I, ↑ATG7	↓LVEDD,↑LVEF, ↓infarct size	[175]
	MIRF^b^	miR-26a	↑ATG7, ↑ATG5, ↑Beclin-1, ↑LC3 II/I ↓USP15, ↓p62	↑Proliferation, ↓Ischemic damage	[176]
	AK139328^b^	miR‐204‐3p	↓ATG7, ↓ATG5, ↓LC3 II/I ↑p62	↓Apoptosis, ↓LVEDD, ↓CK-MB, ↓LDH, ↓LVESD, ↑LVEF, ↑α‐SMA	[177]
	CAIF^b^	p53	↑H_2_O_2_ induced autophagy	↑Myocardin, ↑Apoptosis	[178]
*Myocardial hypertrophy*	MIAT^b^	-	↓LC3	↓p-mTOR, ↓p-AMPK, ↓Ang II-induced MH	[179]
*Ventricular septal defects*	MEG3^b^	miR-7-5p	↑Beclin-1, ↑ATG7, ↓p62	–	[180]
*Immune and Inflammatory diseases*					
*Asthma*	TRPM2-AS^b^	TRPM2	↑LC3	↓Proliferation, ↑Apoptosis, ↓IL-1β, ↓IL-4, ↓IL-6, ↓IL-10, ↓TNF-α, ↓TGF-β	[181]
*CKD*	MANTIS^b^	–	↑Beclin-1, ↑LC3 II/I	↓Migration, ↓Invasion, ↓Proliferation, ↑Apoptosis, ↓Bcl-2, ↑Bax, ↑cle-caspase 3, ↓SOX18	[182]
*COPD*	LINC00987^a^	let-7b-5p	↓LC3 II/I, ↑p62, ↓ATG5	↑Proliferation, ↓Apoptosis, ↓Caspase 3, ↓ROS, ↑SOD1, ↓IL-6, ↓IL-8	[183]
*Lupus nephritis*	HOXA11-OS^b^	miR-124-3p	↓Beclin-1, ↓LC3B	↓Cyr61, ↑Nephrin, ↑Podocin	[184]
*Osteoarthritis*	SNHG7^a^	miR-34a-5p	↓Beclin-1, ↓LC3 II/I	↑Proliferation, ↓Apoptosis ↑SYVN1, ↑PCNA, ↓cle-Caspase-3	[185]
	KLF3-AS1^b^	YBX1	↑LC3 II/I, ↓p62	↓Proliferation, ↑Apoptosis, ↓PI3K, ↓p-Akt, ↓p-mTOR	[186]
	HOTAIR^b^	miR-130a-3p	↑LC3 II/I, ↓p62	↑Proliferation, ↓Apoptosis, ↑Bcl-2, ↓Bax, ↓cle-Caspase-3, ↑Survivin	[187]
	PCGEM1^a^	miR-770	↑ATG12, ↑ATG5, ↑ATG3, ↑Beclin-1	↑Proliferation, ↑BCL2A1, ↑BIRC3, ↑MCL1, ↑Bcl-2, ↓cle- PARP, ↓cle-Caspase 9	[188]
	CIR^b^	-	↓Beclin-1, ↓LC3 II/I	↑COL2A1, ↓MMP-3, ↓Cartilage degeneration, ↑OARSI scores	[189]
	PVT1^b^	miR-27b-3p	↑LC3 II/I, ↑Beclin-1	↑Proliferation, ↓Apoptosis, ↓cle-Caspase 3, ↓IL-6, ↓TNF-α	[190]
	OANCT^b^	FTO	↑Beclin-1, ↑ATG4B, ↓p62	↓M1 polarization, ↓IL-6, ↓TNF-α, ↓IL-12, ↑IL-10, ↑TGF-β1, ↓MMP1, ↓MMP9, ↑Collagen II, ↑Aggrecan	[191]
	NEAT1^a^	miR-122-5p	↑LC3 II/I, ↑Beclin-1	↑Proliferation, ↓Apoptosis, ↑Sesn2, ↑Nrf2, ↑Srx1, ↑Trx1, ↑Ki67, ↓MMP-3, ↓MMP-13, ↑Aggrecan	[192]
	MCM3AP-AS1^b^	miR-149-5p	↑LC3 II/I, ↑Beclin-1	↑Proliferation, ↓Apoptosis, ↓Bax, ↑Bcl-2, ↓cle-Caspase 3, ↓MMP-13, ↑Collagen II, ↑Aggrecan, ↓Notch1	[193]
	GAS5^b^	miR-144	↑LC3 II, ↑Beclin-1	↓Apoptosis, ↓Bax, ↑Bcl-2, ↓mTOR, ↓p-mTOR	[194]
	GAS5^a^	miR-21	↓LC3B, ↓ATG7, ↓Beclin-1	↑Apoptosis, ↑MMP-2, ↑MMP-3, ↑MMP-9, ↑MMP-13, ↑ADAMTS-4	[195]
	POU3F3^a^	miR-29a- 3p	↓Autophagy	↓Chondrocytes injury, ↑Proliferation, ↓Apoptosis	[196]
*Periodontitis*	H19^a^	–	↑LC3 II/I, ↑Beclin-1	↑TNF-α, ↑IL-6, ↓p-AKT	[197]
	FER1L4^a^	–	↑LC3 II/I, ↑Beclin-1	↑FOXO3, ↓p-FOXO3, ↓p-AKT	[198]
*Rheumatoid arthritis*	ZFAS1^b^	miR-2682-5p	↓LC3-II, ↑p62	↓Proliferation, ↑Apoptosis, ↓Bcl-2, ↑Bax, ↑cle-Caspase 3, ↓TNF-α, ↓IL-6, ↑IL-10, ↓ADAMTS9	[199]
*Neurological diseases*					
*Alzheimer’s Disease*	BACE1-AS^b^	miR-214-3p	↓Beclin-1, ↓LC3 II/I, ↑p62	↑Proliferation, ↓Apoptosis, ↓Bax, ↑Bcl-2, ↑Cyclin D1	[284]
	BACE1-AS^b^	miR-214-3p	↓Beclin-1, ↓LC3 II/I, ↑p62, ↓ATG5	↑Proliferation, ↓Apoptosis, ↑Bcl-2, ↓cle-caspase 3, ↑GSH/GSSG ratio	[32]
	17A^b^	–	↑LC3 II/I	↓Apoptosis, ↓Migration, ↓Invasion, ↑G1 phase arrest, ↓Aβ42, ↑GABABR 2,	[200]
	RMRP^b^	miR-3142	↓LC3 II/I, ↓Beclin-1, ↑p62	↑Proliferation, ↓Apoptosis, ↑Bcl-2, ↓Bax, ↓cle-Caspase 3, ↓cle-Caspase 9, ↓TRIB3,	[201]
	MIR600HG^b^	NEDD4L	↑Autophagy	↓Aβ production, ↑Cognitive impairment, ↑PINK1	[202]
	LINC01311^a^	miR-146a-5p	↓Autophagy	↓Apoptosis, ↓APP activity	[203]
*Parkinson Disease*	OIP5-AS1^a^	miR-126	↑Autophagy	↑PLK2, ↓Apoptosis, ↓p-PERK, ↓p-elF2α	[204]
	OIP5-AS1^a^	miR-137	↑Mitochondrial autophagy	↑Proliferation, ↓IL-6, ↑IL-10, ↓IL-1β, CXCL10, ↓CCL-5, ↓G-CSF, ↓CCL4, ↓ROS	[205]
	HOTAIR^b^	miR-874-5p	↓ATG10	↑Proliferation, ↓Apoptosis, ↑Bcl-2, ↓Bax, ↓IL-6, ↓TNF-α, ↓LDH, ↓ROS, ↑SOD	[206]
	NEAT1^b^	–	↓LC3-II/LC3-I	↑Proliferation, ↓PINK1	[207]
	NEAT1^b^	miR-374c-5p	↓LC3-II/I, ↑p62	↑Proliferation, ↓Apoptosis, ↑Dopamine, ↓cle-Caspase 3, ↓cle-PARP, ↑Bcl-2, ↓Bax,	[208]
	NEAT1^b^	miR-107-5p	↓LC3-II/I, ↑p62	↑Proliferation, ↓Apoptosis, ↑Dopamine, ↓cle-Caspase 3, ↑Bcl-2, ↓Bax,	[209]
	SNHG14^b^	miR-519a-3p	↓ATG10	↑Proliferation, ↓Apoptosis, ↓cle-Caspase 3, ↓cle-Caspase 9, ↓LDH, ↓ROS, ↑SOD	[210]
	BDNF-AS^b^	miR-125b-5p	↓LC3-II/I, ↑p62	↑Proliferation, ↓Apoptosis, ↑Dopamine, ↓cle-Caspase 3, ↑Bcl-2, ↓Bax,	[211]
	SNHG1^b^	miR-221/222	↑LC3 II/I	↓Apoptosis, ↓p27, ↓p-mTOR, ↓TH^+^ neuron death	[212]
Others					
*Ischemic stroke*	MIAT^b^	REDD1	↓LC3-II/I, ↑p62	↓REDD1, ↑p-mTOR, ↑Bcl-2, ↓Bax, ↓cle-Caspase 3	[213]
*Spinal Cord injury*	MIAT^a^	RBFOX2	↑MCL-1L, ↑Autophagy,	↓cle-Caspase 3, ↓cle-Caspase 9, ↓Apoptosis, ↓Necrotic tissues	[214]
*Tuberculosis*	DANCR^a^	miR-1301-3p/miR-5194	↑LC3 II/I, ↑ATG4D/ATG5	↑STAT3/STAT5B, ↑RHEB	[215]
	Linc-EPS^b^	–	↑LC3 II/I	↓Apoptosis, ↓Cytochrome c, ↓cle-PARP, ↓cle-Caspase 3, ↑p-JNK	[216]
	MIAT^b^	miR-665	↓LC3-II, ↑p62, ↓Beclin-1	↑Proliferation, ↓Apoptosis, ↓cle-Caspase 3, ↑Bcl-2, ↓Bax	[217]

a: Overexpression; b: knockdown

### LncRNAs in initiation process of autophagy

**Fig. 2:**
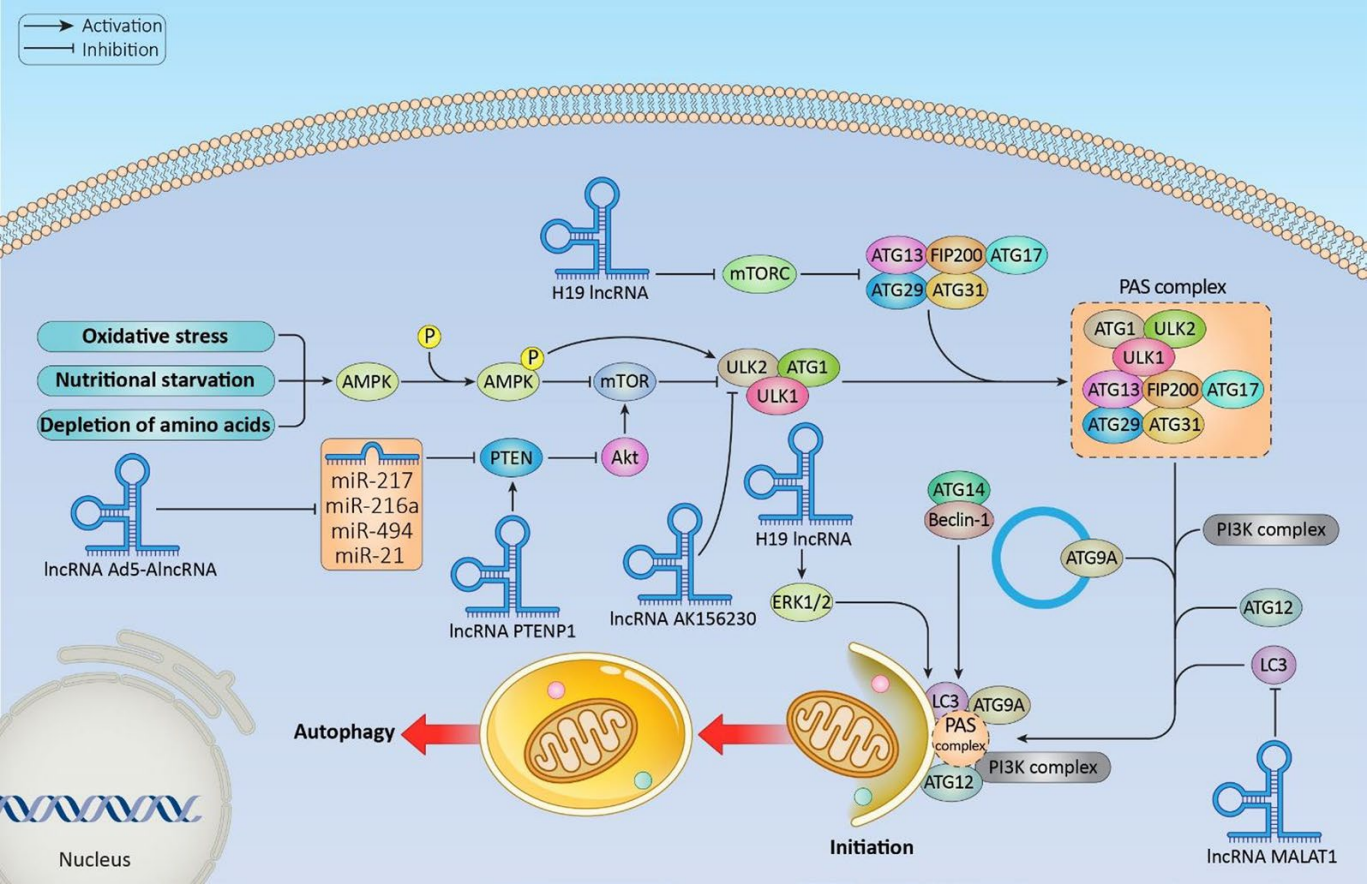
The molecular pathways involving lncRNAs as the initiator of autophagy. In response to energy limitation, autophagy is initiated through the activation of AMPK and inhibition of mTORC1. This process leads to the activation of the ULK1 complex, which includes ULK1, ATG13, FIP200, and ATG101. The expression and activity of these regulatory pathways are influenced by lncRNAs: MEG3 and H19 positively regulate AMPK and mTORC1, while lncRNA AK156230 and Ad5-lncRNA exerts a negative regulatory effect. Furthermore, the collective regulation of the ULK1 complex is modulated by a network of lncRNAs, including PTENP1, MALAT1, TGFB2-OT1, highlighting their roles in fine-tuning the autophagic response under conditions of energy stress

In the course of cellular stimulus such as nutritional starvation, depletion of amino acids, oxidative stress etc., the phosphorylation of AMPK inhibits mTOR leading to the initiation of the autophagic process incited by the activation of ATG1/ULK1/2 complex [218–220]. ATG1/ULK1/2 forms complex with ATG13, FIP200/ATG17, ATG29, and ATG31 to form a scaffold of PAS complex. Then ATG13, and FIP200 interacts with ULK1 targeting to PAS followed by PI3K complex, ATG9A system, ATG-12 conjugation system and LC3-conjugation in a pecking manner involved in the formation of autophagosome, is then transferred to omegasomes (Fig. 2) [221, 222].

Zhou and his group reported the decreased H19 lncRNA expression when treated with high glucose levels inhibited PI3K/AKT/mTOR signaling thereby to the transcriptional activation of DIRAS3 and the H19 knockdown resulted in upregulation of ATG7 and Beclin-1 levels [223]. Further, lncRNA H19 promoted autophagy through the regulation of the DUSP5/ERK1/2 axis [224]. It has been reported that exogenous expression of lncRNA Ad5-AlncRNA resulted in activating autophagy by downregulating various microRNAs (miRNAs) including miR-217, 216a, 494, and 21 that targets PTEN inhibiting the AKT/mTOR pathway [225]. Besides, lncRNA AK156230 represses autophagy by downregulating the ULK2, ATG7, and ATG16/2 expression in mouse embryonic fibroblast cells [226]. Moreover, ectopic expression of lncRNA MALAT1 under oxygen–glucose deprivation/reoxygenation condition resulted in decreased cell death by increasing LC3-II and ULK2 expression as well as by decreasing the p62, and LC3-1 expression along with sponging miR-26b facilitating the brain microvascular endothelial cell autophagy and survival [227]. Further, upregulation of maternally expressed gene 3 (MEG3) triggered autophagy and suppressed tumorigenesis by unmediated interaction with the ATG3 protein, thereby impeding its degradation in ovarian carcinoma [228]. Furthermore, lncRNA PTEN pseudogene-1 (PTENP1) overexpression activated autophagy by increasing the PTEN expression, repressing the PI3K/Akt pathway along with sequestration of the miRNAs including miR-17 and miR-20a that further increases the levels of ATG1, ULK1, and SQSTM1 proteins [229].

### LncRNAs in phagophore nucleation process of autophagy

**Fig. 3:**
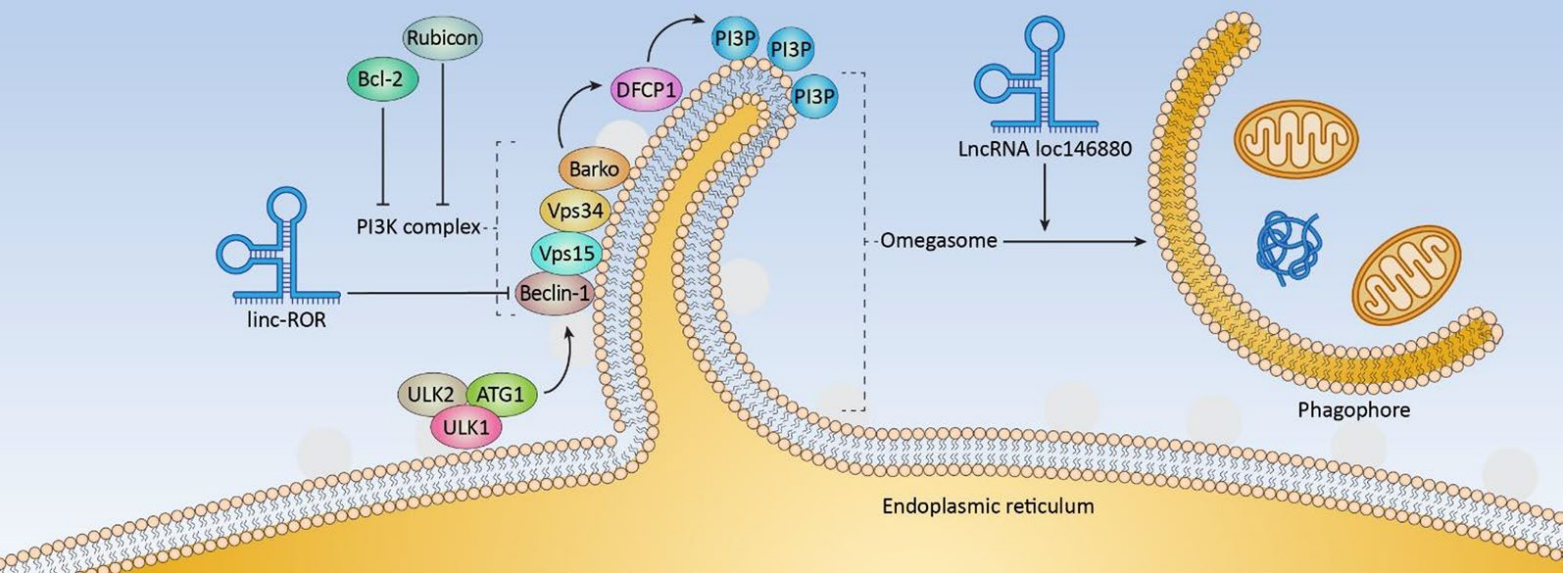
Molecular pathways demonstrating the role of Bcl-2, Rubicon, linc-ROR, and LncRNA loc146880 in autophagy regulation. LncRNAs such as linc-ROR negatively regulates the formation of phagophore nucleation by modulating Beclin-1 levels. Additionally, lncRNA loc146880 is known to modulates the process of phagophore formation

After transferring to omegasomes, ATG1/ULK1 complex forms phosphatidylinositol 3-phosphate (PI3P) by inducing PI3K complex consisting of Vps15, Vps34, Beclin 1, and Barkor, that recruits double FYVE-containing protein 1 (DFCP1) stimulating the omegasome formation [230–232]. It has been reported that Bcl-2 and Rubicon are negatively regulating autophagy by disrupting the class III PI3K complex (Fig. 3) [233, 234].

The lncRNA regulator of reprogramming (linc-ROR) can induces the gemcitabine and tamoxifen resistance and also triggers autophagy by increasing the levels of Beclin-1, however the mechanism between the linc-ROR and Beclin-1 needs to be further explored [235, 236]. A higher expression of the LncRNA loc146880 activated autophagy when treated with PM2.5 and promoted invasion and migration of lung cancer cells (Fig. 3) [237]. Also, lncRNA AC023115.3 increased glycogen synthase kinase-3 (GSK3) expression by downregulating miR-26a and caused apoptosis when treated with cisplatin. Furthermore, lncRNA AC023115.3 chemosensitizes glioma cells by modulating the miR-26a-GSK3β axis [83].

### LncRNAs in autophagosome elongation/closure process

Ubiquitin-like conjugation proteins such as ATG10 (E2-like enzyme) and ATG7 (E1-like enzyme) regulates the ATG12-ATG5-ATG16 complex formation, which facilitates the transformation of LC3B to the membrane-anchored form (LC3-II) from its soluble cytosolic form (LC3-I) [221, 238, 239]. Adaptor proteins like ATG19 and ATG32 along with the neighbor of BRCA1 gene 1 (NBR1), SQSTM1, and Nix, discriminatively promoting the degradation of proteins by binding to LC3-II via attracting them to autophagosomes [221].

Increase in TGFB2 overlapping transcript 1 (TGFB2-OT1) expression triggered by vascular endothelial inflammation upregulated the LARP1 expression and sponging miR-4459 which further increases ATG7, ATG3 and p62 expression [240]. In addition, ectopic overexpression of lncRNA growth arrest specific 5 (GAS5) constrained autophagy by decreasing the levels of ATG5, ATG3, ATG7, ATG12, LC3B, and Beclin-1 expression [195]. Besides, HNF1A-AS1 promoted autophagy by averting the binding of miR-30b to its target genes such as ATG5, ATG12, and Beclin-1 thereby facilitating the HCC tumorigenesis [97]. Moreover, prostate cancer gene expression marker 1 (PCGEM1) lncRNA fostered autophagy by upregulating the ATG5, ATG3, ATG12 and Beclin-1 expression [188]. Further, in hepatocellular carcinoma (HCC) lncRNA HNF1A-AS1 repressed autophagy by preventing the binding of miR-30b-5p to its target protein ATG5 [97]. Interestingly, upregulated expression of lncRNA HOX antisense intergenic RNA (HOTAIR) was observed in HCC, that promotes autophagy by upregulating the levels of ATG7 and ATG3, and negatively regulating miRNAs including miR-34a, miR-10a, miR-331-3p, and miR-454-3p either through recruiting epigenetic modification enzymes by being a scaffold preventing the transcription of miRNA or by capturing miRNAs from their targets [92].

### LncRNAs in autolysosome fusion process

The last step in the autophagy process is the formation of autolysosomes by fusing lysosomes to the autophagosomes degrading the components of the cell. The crucial molecules including membrane proteins of the lysosome such as LAMP1 and LAMP2, Rab-SNARE system, and the adaptor proteins are involved in autolysosome fusion connecting the lysosome to autophagic and endocytic process [221, 241, 242]. Pleckstrin homology domain-containing protein family M member 1 (Plekhm1), an adaptor protein possessing the LC3-interacting region, associates with the homotypic fusion and protein sorting complex mediating the fusion of autophagosomes and endosomes with lysosomes [243]. LncRNA cardiac hypertrophy-associated transcript (Chast) along with Plekhm1 controls the autophagosomes fusion to the lysosomes by decreasing the ATG5 expression [244].

## LncRNAs targeting autophagy in cancers

**Fig. 4:**
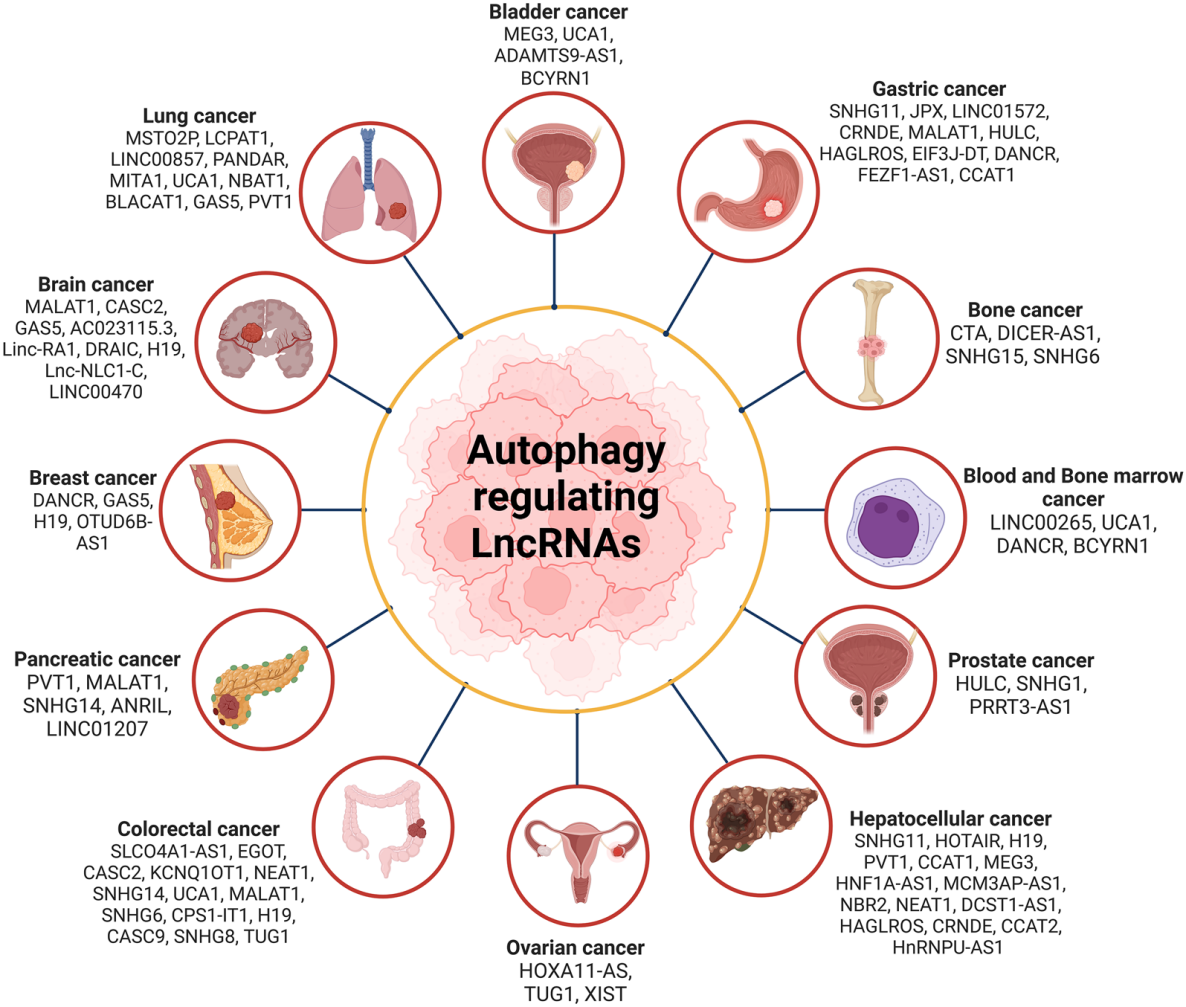
LncRNAs modulating autophagy in various cancers. This figure illustrates the role of lncRNAs as key regulators of autophagy in different types of cancer. lncRNAs modulate gene expression through interactions with miRNAs that are associated with autophagy. Also, they play a crucial role in regulating several hallmarks of cancer, including uncontrolled proliferation, enhanced survival, invasion, migration, epithelial-to-mesenchymal transition (EMT), and angiogenesis. These lncRNA-mediated modulations of autophagy contribute to cancer progression, highlighting their potential as therapeutic targets

Cancer is one of the most significant health menaces of this century with approximately 19.3 million new incidences and 10 million deaths worldwide [245]. Although different treatment regimens have ameliorated the quality of life and survival patients, still outcomes at the advanced stages are dismal [4, 246, 247]. With the increasing demand for safe and efficacious treatments to increase the quality of life in patients, it has become necessary to understand and recognize the causative features and develop diagnostic and therapeutic interventions to circumvent this multigenic disease [4, 246, 248–252]. Growing lines of evidence implicates lncRNAs to be associated with modulating various hallmarks of cancer either by acting as tumor suppressor or oncogenic elements [253]. LncRNAs being the master regulators have been known to regulate the autophagic process by acting as ceRNAs to sequester the autophagy related miRNAs involved in cancer progression (Fig. 4).

Various studies have shown the mechanistic action of lncRNAs that are involved in cancer development through modulating autophagy [36, 37, 48, 50, 57, 66, 71, 83, 89, 98, 106, 111, 112, 120, 129, 137, 138, 140]. For instance, Zhang and the group demonstrated the role and mechanism of the lncRNA LINC00265 in acute myeloid leukemia (AML). In this study, the expression of LINC00265 was increased in AML cells and modulated the Interferon regulatory factor 2 (IRF2) expression via acting as ceRNAs for miR-485-5p leading to upregulation of autophagy [41]. In another study, it was found that lncRNA urothelial carcinoma-associated 1 (UCA1) promoted autophagy by regulating the expression of ATG7 via targeting the miR-96-5p levels [42]. LncRNA differentiation antagonizing non-protein coding RNA (DANCR) was observed as an oncogene in AML by regulating autophagy and chemoresistance. It was found that DANCR through its regulation of miR-874-3p/ATG16L1 axis conferred cytarabine resistance and targeting this lncRNA might be a novel approach for combating AML [43].

Due to its heterogeneity and stratified subtypes, breast cancer reports for the highest incidences of cancer occurring worldwide in the year 2020 [245]. Although various treatment approaches have been devised, none of them have been efficient to completely eradicate this malady at the advanced stage [254]. LncRNAs through its post transcriptional and translational regulation hold promise in the diagnosis and treatment of breast cancer. For instance, Zhang and coworkers probed the mechanism of DANCR in modulation of apoptosis and autophagy in breast cancer. Knockdown of DANCR resulted in increased expression of different apoptosis and autophagic markers such as caspase-3, caspase-9, Bax/Bcl-2, LC3B, Atg5. This inhibitory and anti-cancer effect of DANCR was found to be mediated by targeting PAX6 expression via sponging miR-758-3p in breast cancer cells [44]. In another study, overexpression of lncRNA growth arrest-specific 5 (GAS5) was found to induce autophagy by regulating the levels of unc-51 like autophagy activating kinase (ULK)1/2. Further, GAS5 was shown to promote cisplatin chemosensitivity in breast cancer cells by mediating the ULK1/2 levels [45]. Another study assessed the role of lncRNA H19 regulation in tamoxifen-resistant breast cancer. Silencing of H19 resulted in inhibition of autophagy by increasing the binding of DNMT3B in the promoter region of Beclin-1 [46]. Another study found that lncRNA OTUD6B-AS1 was a negative regulator of DNA damage response in breast cancer by inhibiting the activation of phosphorylated forms of ATM, RAD51, and ATR [47].

Colorectal Cancer (CRC) is a heterogeneous malignancy causing approximately 6,00,000 deaths annually worldwide [245]. Apart from early intervention by surgical resection, chemotherapy, radiotherapy, or a combination of all, no effective treatment exists for advanced CRC. Recently, lncRNAs have emerged as major mediators of autophagy in CRC [54, 57, 60, 61, 255, 256]. For instance, in a study, it was observed that lncRNA CASC2 was downregulated in colon cancer cell and tissues, and overexpression of CASC2 resulted in growth inhibition with induction of apoptosis and autophagy by increasing the levels of LC3B II and Beclin-1 [55]. In another study, lncRNA eosinophil granule ontogeny transcript (EGOT) expression was high in clinical samples of CRC. Ectopic expression of EGOT increased the proliferation and invasive attributes of CRC cells by inhibiting the apoptosis and autophagy and decreasing the expression of Beclin-1, LC3B II, BAX and cleaved caspase-3 [54]. Another study revealed the functional role of lncRNA nuclear paraspeckle assembly transcript 1 (NEAT1) in inducing the hallmarks in CRC cell lines. It was demonstrated that the proliferation of CRC cells was markedly decreased by NEAT1 knockdown, which also improved 5-FU sensitivity. Further, NEAT1 knockdown also inhibited the expression of Beclin-1, ULK1, and the ratio of LC3-II/I in CRC cell lines via targeting miR-34a [57]. Wang and colleagues sought to identify the biological functions of lncRNA SLCO4A1-AS1 in CRC. SLCO4A1-AS1 modulated the expression of partition-defective 3 (PARD3) by acting as ceRNA for miR-508-3p leading to upregulation of proliferation in CRC. Moreover, knockdown of SLCO4A1-AS1 resulted in attenuated autophagy and proliferation with increased apoptosis in CRC cell lines [58]. Another study found that lncRNA MALAT1 was upregulated in CRC tissues. Further, the expression of LC3-II mRNA was correlated with the MALAT1 level. Furthermore, MALAT1 also significantly increased cell proliferation and activated autophagy while decreasing apoptosis in CRC cells via sponging miR-101 [60]. LncRNA small nucleolar RNA host gene 14 (SNHG14) and ATG14 was found to be upregulated in clinical CRC samples. Further, miR-184 was found to be a direct target of SNHG14, and miR-184 modulated the expression of ATG14. Overexpression of SNHG14 led to increased proliferation, migration, invasion, and cisplatin resistance in CRC cell lines [34]. Another study, it was observed that lncRNA taurine up-regulated gene 1 (TUG1) targeted miR-195-5p by modulating HDGF levels, thereby regulating miR-195-5p/HDGF/DDX5/β-catenin axis in CRC resistance [66].

Additionally, it’s essential to note the interplay between lncRNAs, autophagy, and epithelial-mesenchymal transition (EMT) in cancer. EMT is a biological process that allows epithelial cells to undergo multiple biochemical changes enabling them to assume a mesenchymal cell phenotype, which includes enhanced migratory capacity, invasiveness, and resistance to apoptosis, contributing to cancer progression [257, 258]. The role of lncRNA HOTAIR as a critical autophagy regulator has been discussed. However, it also functions as a scaffold for chromatin modifiers, such as PRC2 and LSD1, thus driving the transcriptional reprogramming that fosters the transition from epithelial to mesenchymal traits [259]. Another example is the lncRNA MALAT1, which in addition to induce autophagy, it has been reported to regulate and EMT in esophageal cancer by affecting the Ezh2-Notch1 signaling pathway [260], serving as a linker between these two pivotal processes. Therefore, both HOTAIR and MALAT1, as well as probably other lncRNAs, may serve as a crucial linker between autophagy and EMT, orchestrating complex regulatory networks that facilitate cancer progression and resistance to therapy. Understanding the link between lncRNAs, autophagy, and EMT can potentially provide insights into developing novel therapeutic strategies for cancer treatment.

## LncRNAs modulating autophagy in cardiovascular diseases

**Fig. 5:**
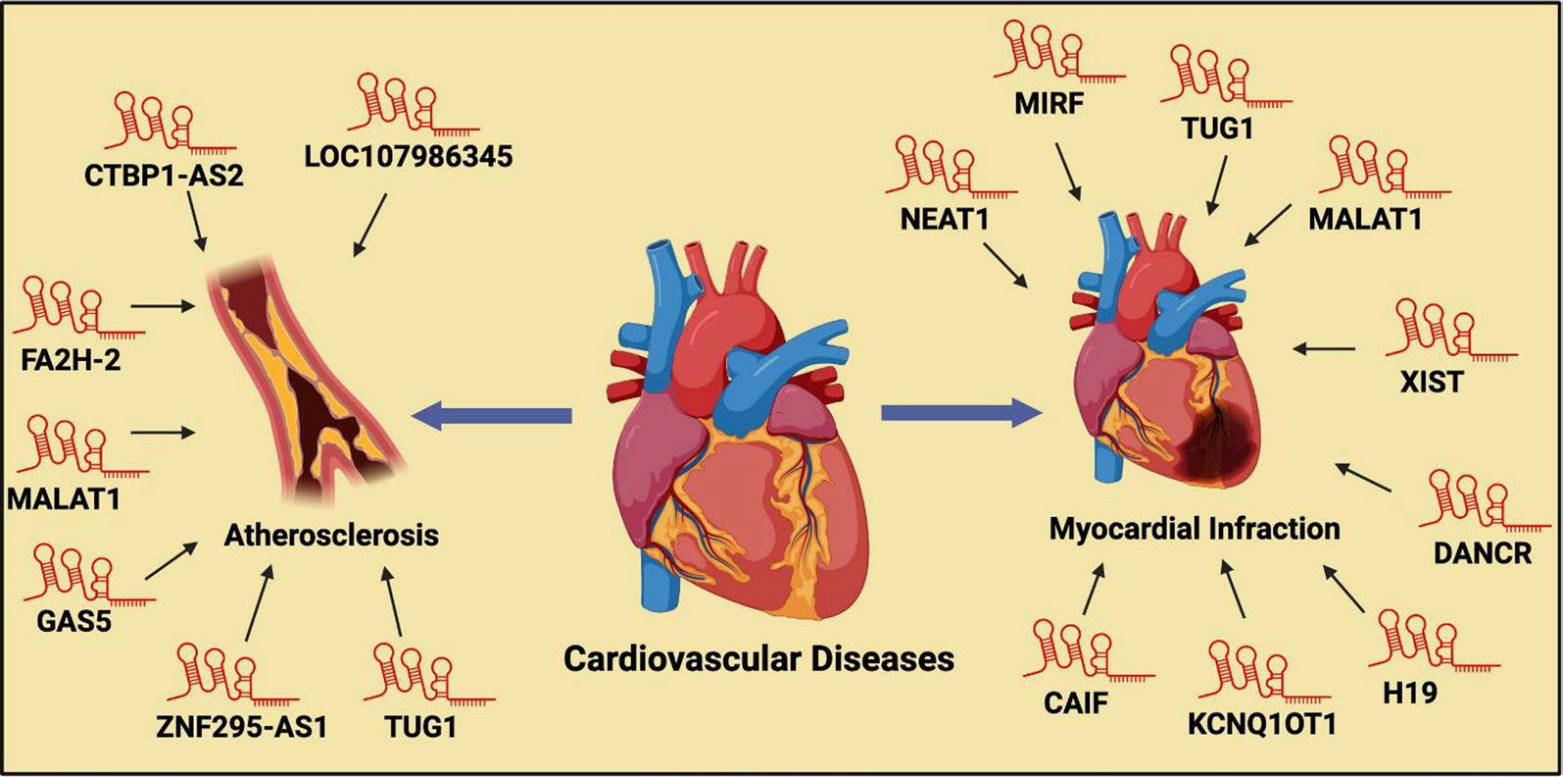
Regulation of autophagy by lncRNAs in cardiovascular diseases. This figure depicts the role of various lncRNAs in the modulation of autophagy and their involvement in cardiovascular disease pathogenesis. In the context of atherosclerosis, lncRNAs such as MALAT1, GAS5, TUG1, CTBP1-AS2, FA2H-2, ZNF295-AS1, and LOC107986345 have been identified as key regulators of autophagic processes, influencing disease development and progression. Similarly, in myocardial infarction, lncRNAs including CAIF, H19, DANCR, XIST, NEAT1, and MIRF are implicated in the regulation of autophagy, contributing to the etiology of the disease. This network of lncRNAs underline their critical role in modulating autophagic pathways and their potential impact on cardiovascular disease mechanisms

Cardiovascular diseases have emerged as a global health epidemic over the past few decades, affecting a significant proportion of the population in both developed and developing nations [3, 261]. The etiological factors contributing to cardiovascular diseases are multifaceted, including both external and internal influences such as elevated cholesterol levels, excessive alcohol consumption, poor dietary habits (e.g., insufficient intake of fruits), obesity, diabetes, hypertension, genetic predisposition, and sedentary lifestyles [261]. Autophagy dysfunction has been implicated in various cardiovascular diseases, including atherosclerosis, myocardial infarction, and heart failure. Dysregulated autophagy adversely impacts cardiac cell viability, intracellular protein quality control, and the regulation of inflammatory and oxidative stress responses. Impaired autophagy leads to the accumulation of damaged organelles and proteins, resulting in cardiomyocyte dysfunction and pathological cardiac remodeling [262–265]. Recent studies have identified lncRNAs as critical modulators of autophagy dysfunction in several cardiovascular diseases, highlighting their potential as therapeutic targets for mitigating disease progression (Fig. 5).

Myocardial infarction (MI), commonly referred to as a heart attack, is characterized by the necrosis of cardiac muscle tissue due to reduced or complete cessation of blood flow to the heart, typically caused by a blockage or thrombus in the epicardial arteries [266]. Dysregulated macroautophagy has been implicated in exacerbating myocardial injury during MI, with lncRNAs playing a critical role in modulating autophagy in cardiac cells [267]. A study identified the lncRNA autophagy promoting factor (APF) as a key regulator of autophagy in cardiomyocytes by targeting miR-188-3p and ATG7, influencing the autophagic response and the progression of myocardial infarction [174]. Another study demonstrated that lncRNA AK088388 interacts with miR-30a, resulting in elevated Beclin-1 expression and increased autophagy in cardiomyocytes, contributing to cell damage [171]. Liu et al. reported that lncRNA cardiac autophagy inhibitory factor (CAIF) regulates autophagy in cardiomyocytes through its control of p53 and myocardin expression, further elucidating the complex interplay between lncRNAs and autophagic pathways during myocardial infarction [178].

Another lncRNA), known as cardiac hypertrophy-related factor (CHRF), has been shown to exacerbate myocardial ischemia/reperfusion (I/R) injury by promoting autophagy in vitro through targeting ATG7 [268]. A study demonstrated that inhibition of the lncRNA hypoxia/reoxygenation injury-related factor in myocytes (HRIM) improved cardiomyocyte viability by reducing autophagy levels [269]. Liang et al. found that overexpression of miR-26a attenuated ischemia-induced cell death by enhancing autophagy via targeting Usp15 (ubiquitin-specific peptidase 15). Further analysis revealed that the lncRNA myocardial infarction-regulatory factor (Mirf) modulates miR-26a and inhibits autophagy, contributing to the regulation of myocardial cell survival during ischemic stress [176]. The lncRNA MALAT1 has been shown to influence myocardial infarction-related events through three distinct mechanisms. Firstly, MALAT1 promotes cardiomyocyte injury by sponging miR-20b and enhancing Beclin1-mediated autophagy. Secondly, MALAT1 protects cardiomyocytes from isoproterenol-induced apoptosis by sponging miR-558 and upregulating autophagy via the ULK1 pathway. Lastly, MALAT1 inhibits autophagy by modulating the TSC2-mTOR pathway, which in turn promotes apoptosis in cardiomyocytes [159, 160, 162]. In another study, the lncRNA TUG1 (taurine upregulated gene 1) was found to target miR-142-3p, contributing to the induction of apoptosis through autophagy in ischemia/hypoxia-challenged cardiomyocytes by upregulating HMGB1 and Rac1 expression [169]. Similarly, inhibition of the lncRNA XIST (X-inactive specific transcript) was shown to ameliorate myocardial ischemia/reperfusion (I/R) injury by targeting miR-133a, suppressing autophagy, and regulating SOCS2 expression [165]. Collectively, these findings, along with numerous other studies, highlights the critical role of lncRNAs in modulating autophagy and impacting the pathophysiology of MI [161, 166, 172, 173, 175–179, 270].

Diabetic cardiomyopathy is a heart disease induced by diabetes mellitus (DM) that ultimately leads to heart failure [271]. Non-coding RNAs, particularly miRNAs and lncRNAs, have been found to significantly influence myocardial conditions in diabetic patients [272]. It is known that diabetic cardiomyopathy is particularly sensitive to alterations in lncRNA expression. For instance, in a study, it was demonstrated that knockdown of lncRNA AK139328 in cardiomyocytes suppressed autophagy and prevented apoptosis [177]. Further, the lncRNA diabetic cardiomyopathy-related factor (DCRF) has been identified as a key regulator of autophagy in diabetic cardiomyopathy. DCRF is highly expressed under diabetic conditions and sponges miR-551b-5p, leading to elevated PCDH17 levels and enhanced autophagy in glucose-treated cardiomyocytes [273]. In a more recent study, lncRNA GAS5 was reported to reverse the inhibition of autophagy in the myocardial cells of diabetic rats [274]. Moreover, another study found that lncRNA H19 suppresses autophagy in cardiomyocytes; knockdown of H19 promoted autophagy by upregulating the expression of DIRAS3 [223]. These findings emphasize the critical role of lncRNAs in modulating autophagy and apoptosis in the context of diabetic cardiomyopathy.

## LncRNAs modulating autophagy in immune and inflammatory related diseases

**Fig. 6:**
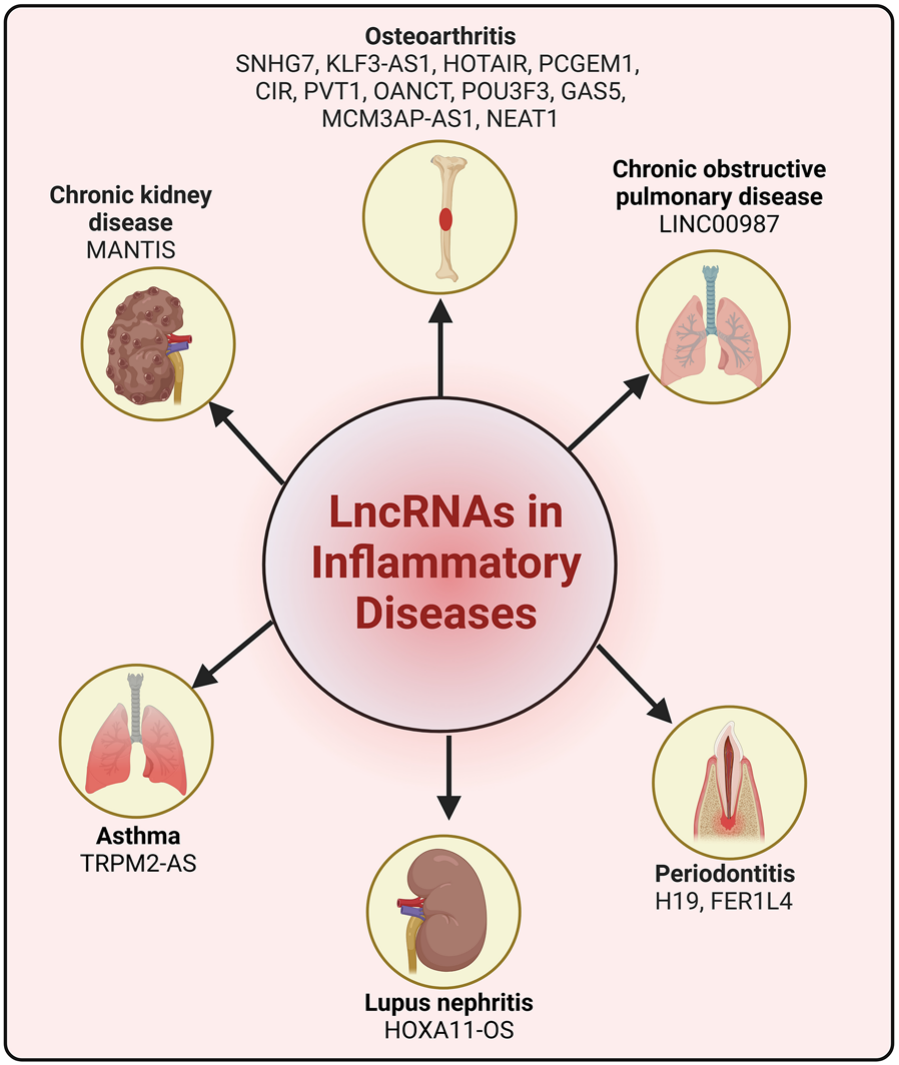
LncRNAs regulating autophagy in various inflammatory diseases. Various lncRNAs are known to modulate the autophagy in different inflammatory diseases such as osteoarthritis, asthma, periodontitis, lupus nephritis, chronic kidney and pulmonary diseases. These lncRNAs exert their influence on key autophagic processes such as autophagosome formation, lysosomal function, and the degradation of misfolded proteins. This figure highlights the intricate roles of lncRNAs in modulating autophagic flux, exhibiting their potential as therapeutic targets for the treatment of inflammatory related diseases

Autophagy is a vital cellular process involved in regulating immune responses and maintaining inflammatory homeostasis. Defective autophagy has been implicated in the pathogenesis of several autoimmune and chronic inflammatory diseases, including asthma, osteoarthritis, chronic kidney disease, systemic lupus erythematosus, and Crohn’s disease. Impaired autophagy can lead to the accumulation of damaged cellular components, thereby activating inflammatory signaling pathways and causing immune dysregulation [275, 276]. Targeting autophagy-related pathways has emerged as a promising therapeutic strategy for the management of chronic inflammatory and autoimmune conditions (Fig. 6).

Osteoarthritis (OA) is one of the most prevalent chronic musculoskeletal diseases, significantly impacting patients’ quality of life. Therefore, understanding the underlying biological mechanisms is essential for improving the diagnosis and treatment of OA [277, 278]. LncRNAs, which play a crucial role in regulating various genes, have been found to influence the progression of osteoarthritis by modulating autophagy in chondrocytes [185, 186, 188, 190, 192, 193]. A 2018 study reported that lncRNA-CIR was overexpressed in OA patients, positively regulating autophagic genes such as LC3B-I/II and Beclin-1, while negatively affecting the overall progression of OA [189]. Additionally, research by Song et al. demonstrated that lncRNA-GAS5 increased chondrocyte apoptosis and decreased autophagy by downregulating autophagy-related genes, including ATG-7, LC3B, and Beclin-1 [195]. This same lncRNA was also found to interact with miR-144, ultimately regulating the expression of mTOR [194]. Furthermore, the lncRNA HOTAIR has been shown to promote apoptosis and suppress autophagy in chondrocytes in knee OA [187]. These findings provide valuable insights into the molecular mechanisms of OA and have the potential to contribute to the development of novel therapeutic strategies for treating this disease.

In addition to osteoarthritis, LncRNAs have been shown to influence autophagy in various other inflammatory conditions [181, 279]. In 2019, Yu TX et al. reported that overexpression of the lncRNA H19 can inhibit autophagy in mucositis [280]. Similarly, upregulation of another lncRNA, MANTIS, was found to inhibit autophagy in HUVECs injury in chronic kidney disease [182]. Additionally, lncRNA LINC00987 was shown to suppress autophagy in BEAS-2B cells during studies of chronic obstructive pulmonary disease [183]. Conversely, lncRNAs have also been observed to promote autophagy in the context of periodontitis. For example, the lncRNA FER1L4 was found to induce autophagy in periodontal ligament stem cells under external compressive force [198]. Similar pro-autophagic effects were observed for lncRNA H19 in periodontitis [197]. These findings highlights the importance of understanding the complex regulatory roles of lncRNAs in autophagy, which could lead to the development of novel therapeutic strategies for treating a wide range of inflammatory diseases.

## LncRNAs modulating autophagy in neurological diseases

**Fig. 7:**
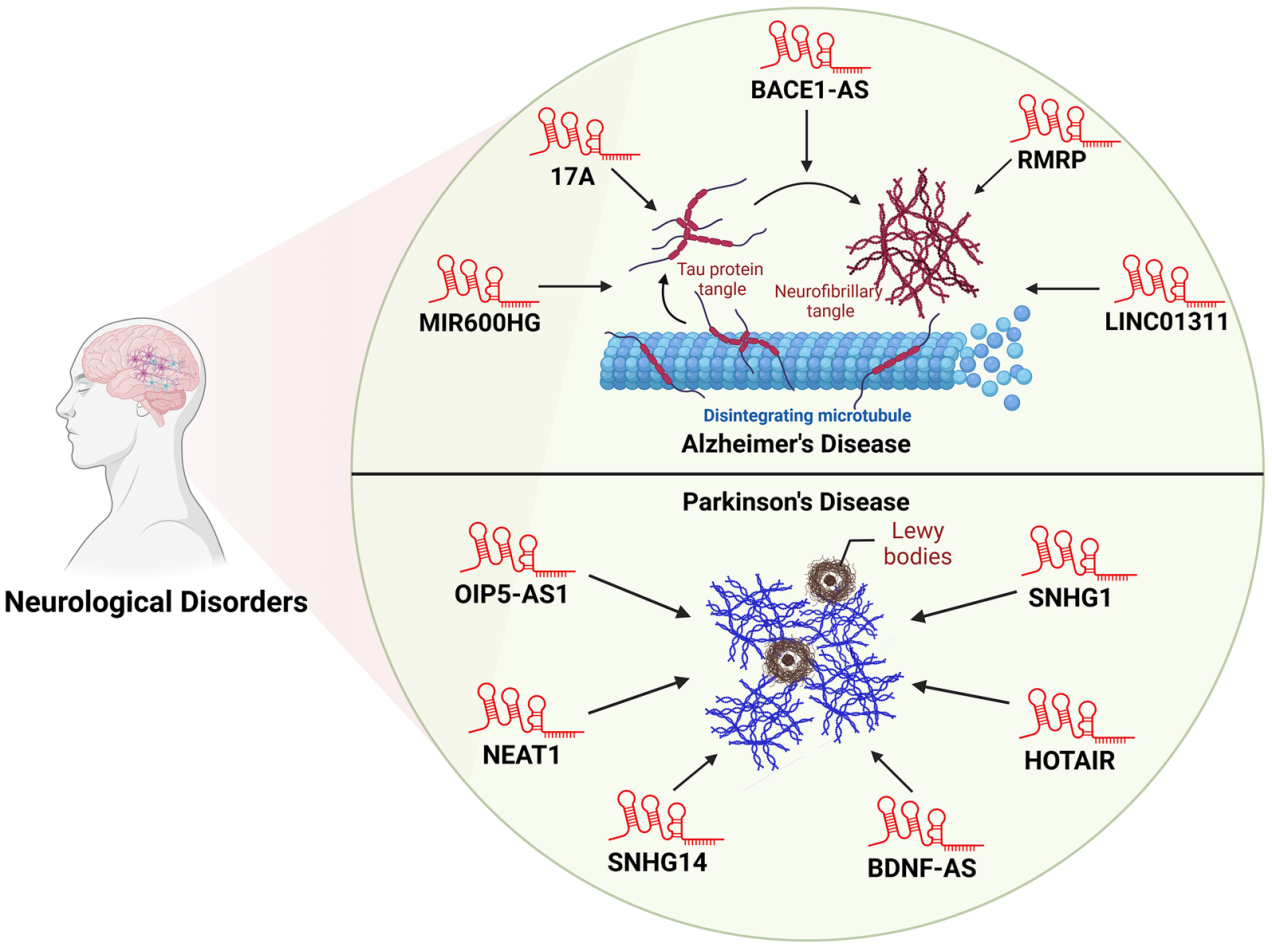
Autophagy-regulating lncRNAs in neurological diseases. Long non-coding RNAs (lncRNAs) have emerged as key modulators of autophagy in neurodegenerative disorders, particularly Alzheimer’s and Parkinson’s diseases. In Alzheimer’s disease, lncRNAs such as BACE1-AS, MIR600HG, 17A, RMRP, and LINC01311 have been implicated in the regulation of autophagy-related pathways, influencing the progression of the disease. Similarly, in Parkinson’s disease, lncRNAs including OIP5-AS1, NEAT1, HOTAIR, SNHG1, and BDNF-AS are involved in the regulation of autophagy genes, contributing to neuronal dysfunction and disease pathology

Autophagy is an important component in preserving cellular processes by eliminating damaged proteins and organelles. Accumulating evidence has implicated the deregulation of autophagy in the pathogenesis of various neurological diseases, including Alzheimer’s, Parkinson’s, and Huntington’s diseases [21, 22]. Recent studies have revealed the association of lncRNAs in modulating autophagy pathways, providing new insights into the molecular mechanisms underlying neurological disorders (Fig. 7).

Alzheimer’s disease is recognized as the most severe and significant cause of dementia in the aging population, posing a major public health concern, particularly in countries with a high proportion of elderly individuals. The etiology of Alzheimer’s disease remains contentious; however, it is generally attributed to a combination of genetic and environmental factors [281, 282]. Recent studies have found lncRNAs to be linked with Alzheimer’s disease and they have also been stated to affect autophagy in disease models and cell lines [201–203]. For instance, the lncRNA 17A has been reported to inhibit autophagy when overexpressed, whereas its knockdown results in enhanced autophagy [200]. Similarly, lncRNA NEAT1 has been shown to inhibit PINK1-dependent autophagy by promoting NEDD4L-mediated degradation of PINK1 [283]. Additionally, another lncRNA, BACE1-AS, was found to exacerbate Aβ1–42-induced cellular injury in Alzheimer’s disease by upregulating autophagy. This effect was mediated by its function as a ceRNA for miR-214-3p, thereby influencing the expression of ATG5 [32]. These findings provide valuable insights into the molecular mechanisms underlying Alzheimer’s disease and may open new avenues for future research aimed at understanding the disease’s pathogenesis, as well as developing novel preventive and therapeutic strategies. Further, lncRNA BACE1-AS was found to modulate the levels of miR-214-3p, thereby regulating the isoflurane-induced neurotoxicity in Alzheimer’s disease [284].

Approximately 1% of individuals over the age of 60 are affected by Parkinson’s disease globally. The etiology of Parkinson’s disease is attributed to a combination of aging, genetic predisposition, and environmental factors [285, 286]. LncRNAs are crucial in brain development and synapse formation, and a study has shown that over 87 lncRNAs exhibit significantly altered expression levels in patients with Parkinson’s disease. This differential expression impacts autophagy in neuronal cells as well [287]. The lncRNA BDNF-AS has been shown to enhance MPP + -induced autophagy in Parkinson disease cell model by targeting miR-125b-5p [211]. Similarly, the lncRNAs HOTAIR and NEAT1 also promoted autophagy in Parkinson disease cell models[206, 207]. In contrast to these autophagy enhancers, Qian et al. identified the lncRNA SNHG1, an autophagy suppressor, where its downregulation led to increased autophagy in Parkinson disease cell model [212]. Further, the lncRNA OIP5-AS1 indirectly regulated autophagy through the modulation of PLK2/α-synuclein interactions [204]. Given their significant impact on brain function, lncRNAs are key to understand the pathogenesis and progression of Parkinson’s disease [205, 208–210, 212]. The insights gained from studying these lncRNAs could inform strategies for the prevention and treatment of Parkinson’s disease. Understanding the intricate relationship between autophagy and neurological disorders presents opportunities for developing innovative therapeutic strategies aimed at restoring autophagy functionality and mitigating disease-related pathology. Further research is necessary to elucidate the specific mechanisms underlying autophagy dysregulation and to translate these findings into effective therapeutic interventions for neurological disorders.

## LncRNAs modulating autophagy in other diseases

LncRNAs recognized as master regulators, play a crucial role in the autophagy processes involved in various diseases. Tuberculosis (TB), which affects approximately 10 million people annually, is one of the most devastating infectious diseases worldwide [288]. Research has indicated that the autophagy of macrophages is a key factor in the pathogenesis of Mycobacterium tuberculosis (MTB) [289] making lncRNAs, which regulate autophagy, a focus of recent studies aimed at understanding TB pathophysiology [215, 217]. For example, a 2019 study by Li M. et al. demonstrated that the downregulation of lncRNA PCED1B-AS1 led to reduced apoptosis and enhanced autophagy in macrophages during TB [290]. Similarly, downregulation of lincRNA EPS had comparable effects on RAW264.7 macrophages [216]. Given the critical role of macrophages in eliminating the TB bacterium, these findings could be instrumental in developing novel therapeutic strategies against TB. LncRNAs are also increasingly recognized for their roles in liver diseases, including hepatitis C and nonalcoholic fatty liver disease (NAFLD), both of which are of significant concern due to their complex pathogenesis and severe prognosis. For instance, a study by Ferrasi et al. identified a distinctive lncRNA expression profile in hepatic tissues at various stages of fibrosis and hepatocellular carcinoma (HCC), revealing novel tumor suppressor lncRNAs as potential diagnostic markers and therapeutic targets in early liver injury and HCC development, particularly in the context of hepatitis C infection [291]. Moreover, recent research on the lncRNA Neat1 in liver fibrosis has observed the upregulation of Neat1 in fibrotic liver tissues with the activation of hepatic stellate cells, which are central to fibrosis [292]. Neat1 modulates cytohesin 3 expression by sponging miR-148a-3p and miR-22-3p, offering insights into liver fibrogenesis and potential lncRNA-directed therapies for liver fibrosis. In the context of NAFLD, a pivotal study identified four autophagy-related lncRNAs—PSMG3-AS1, MIRLET7BHG, RP11-136K7.2, and LINC00925—as key components in the complex network of ceRNAs. These lncRNAs interact within cellular RNA networks to regulate gene expression, influencing the progression of NAFLD and providing new directions for research and therapeutic strategies [293]. Additionally, research on chronic pancreatitis has highlighted the role of Lnc-PFAR in the activation of pancreatic stellate cells and pancreatic fibrosis via RB1CC1-induced autophagy, suggesting its potential as a therapeutic target. [294]. Collectively, these studies enhance our understanding of how lncRNAs modulate autophagy across various diseases, not only broadening our knowledge of their regulatory functions but also opening new avenues for targeted therapeutic interventions.

## Clinical implications of lncRNAs

LncRNAs have emerged as pivotal elements in modern medicine, transcending their basic cellular roles to become central in both therapeutic and diagnostic applications. Their involvement in diverse diseases as accurately described in this review, ranging from cancer to neurological disorders, emphasize their unique potential in personalized medicine. Current clinical studies and trials are examining the role of lncRNAs not only as diagnostic markers but also as therapeutic agents capable of modifying disease outcomes. Significantly, lncRNAs have been linked to the regulation of autophagy, a cellular process crucial for maintaining cellular homeostasis and responding to stress. This connection suggests their influential role in disease progression and treatment outcomes. The dual functionality of lncRNAs in diagnostics, as biomarkers for early disease detection, and in therapeutics, as targets or agents in disease management, highlights their versatility and the necessity for ongoing research to harness their full clinical potential.

### LncRNAs in the clinical management of cancer

The clinical landscape of cancer diagnosis and therapy is increasingly recognizing the crucial role of lncRNAs. As biomarkers, lncRNAs like H19 and PCA3 are gaining prominence for their predictive accuracy in early cancer detection and prognosis assessment [295, 296]. Other studies have shown that the presence of DSCAM-AS1, and GATA3-AS1 correlates with disease progression in breast cancer, offering valuable insights for personalized treatment strategies [297]. Furthermore, the therapeutic applications of lncRNAs are advancing, with several entering clinical trials to evaluate their efficacy in targeted therapy protocols. For instance, targeting lncRNA H19 has shown promising results in reducing tumor growth and metastasis in anaplastic thyroid cancer, highlighting its potential as a clinical molecular therapy target [298]. Similarly, the association of lncRNA MALAT1 with tumor growth and metastasis in colorectal cancer positions it as both a diagnostic marker and a therapeutic target, reflecting its dual role in cancer management [299, 300]. Beside the relevant insights underlying the potential of lncRNAs for cancer treatment, they have been actively explored in clinical trials, marking a significant transition towards more targeted treatments. For example, the clinical trial [301] investigates the diagnostic capabilities of lncRNA MFI2-AS1 in kidney cancer, aiming to establish it as a reliable biomarker for early detection and disease progression. Similarly, [302] examines lncRNAs WRAP53 and UCA-1 in hepatocellular carcinoma as potential diagnostic biomarkers. UCA-1 is a well-documented lncRNA that is overexpressed in several cancers, including bladder and breast cancers. It promotes tumor growth, metastasis, and chemotherapy resistance by modulating key signaling pathways and gene expression. Clinically, UCA-1 serves as a potential biomarker for diagnosis and prognosis, particularly in urothelial carcinoma where it can help in distinguishing malignant from benign conditions [303]. The role of UCA-1 in conferring drug resistance also presents a unique opportunity for targeted therapy, where downregulating UCA-1 could enhance the responsiveness of tumors to chemotherapy [304, 305]. Another notable trial, [306], evaluates the safety and efficacy of INT-1B3, an RNA mimic agent, in treating advanced solid tumors. These mentioned clinical trials highlight the evolving role of lncRNAs in precision oncology, offering promising new avenues for cancer diagnosis and treatment.

### LncRNAs in the clinical management of cardiovascular diseases

In cardiovascular medicine, lncRNAs are gaining recognition as valuable diagnostic biomarkers and therapeutic agents. They have been shown to play a crucial role in diagnosing conditions like heart failure, acute myocardial infarction (AMI), and diabetic cardiomyopathy. Emerging studies highlight the potential of lncRNAs to provide new therapeutic approaches by targeting specific pathways involved in cardiac repair and regeneration [307, 308]. For instance, lncRNA-NRF levels were significantly higher in patients with heart failure demonstrating its high diagnostic potential as biomarker for heart failure post-AMI [309]. On the other side, a study by Gonzalo-Calvo et al. identified other lncRNAs like LIPCAR, MIAT, and SENCR with considerable potential in diagnosing diabetic cardiomyopathy [310]. From a therapeutic point of view, ncRNA-targeted treatments are making strides in cardiovascular diseases and show potential in moving from animal models to human clinical applications. Clinical trials such as [311] and [312] are exploring miRNA-based strategies for heart failure. In hyperlipidaemia, inclisiran, a kind of ncRNA targeting PCKS9, demonstrated significant LDL cholesterol reduction in the ORION trials [313], showcasing the therapeutic potential of lncRNA-targeted treatments in cardiovascular conditions. Clinical trials are now exploring how lncRNA-targeted therapies could reduce myocardial injury and improve recovery [314, 315]. In this context, lncRNA CoroMarker has been specifically identified and used as a biomarker for diagnosing coronary artery disease (CAD). It has been tested in clinical settings and has shown to provide higher sensitivity and specificity compared to traditional biomarkers for CAD, making it a valuable tool for early diagnosis and management of this condition [316]. Furthermore, MIAT has been studied as a biomarker for predicting the risk of myocardial infarction. Its expression levels are significantly altered in patients following a myocardial infarction, and it has potential clinical applications in assessing patient risk and guiding treatment strategies to prevent heart failure post-infarction [317].

### Clinical applications of lncRNA in neurological and other diseases

LncRNAs hold significant promise as diagnostic markers in the context of neurological diseases, particularly in Alzheimer’s and Parkinson’s diseases. Their role extends to therapeutic applications, where modulation of specific lncRNAs has been linked to improved outcomes in neurodegenerative disorders. Through high-throughput analysis, Huaying et al. revealed a signature of five lncRNAs closely associated with Alzheimer’s disease progression showing promise as potential biomarkers for early diagnosis of the disease [318]. In a similar frame, Firat et al. identified novel panels of brain-enriched lncRNAs, differentially expressed in patients, as potential biomarkers for early Alzheimer’s disease and other dementias [319]. Furthermore, Feng et al. found that the plasma level of lncRNA BACE1 were significantly higher in Alzheimer’s disease patients suggesting its utility as a diagnostic tool with high specificity [320]. Similarly, Simchovitz et al. found lncRNA LINC-PINT in elevated levels in the substantia nigra of patients affect by Parkinson’s disease, suggesting its involvement in neurodegenerative processes and potential as a biomarker [321].

Beyond neurological conditions, lncRNAs are emerging as crucial in diagnosing and clinical monitoring in liver diseases. A comprehensive review by Zeng et al. highlights the emerging role of lncRNAs like NEAT1, MEG3, MALAT1 as non-invasive biomarkers in NAFLD [322]. In the same frame, Shoraka et al. investigated the clinical potential of lncRNA-ATB in hepatitis B virus (HBV)-related cirrhosis and chronic hepatitis B (CHB). They revealed that elevated plasma levels of lncRNA-ATB are significantly associated with HBV-related cirrhosis, demonstrating its specificity as a biomarker for this condition [323]. Additionally, the same study also finds that lncRNA-ATB levels are lower in non-cirrhotic CHB patients compared to healthy controls, suggesting its sensitivity in diagnosing CHB. This highlights the great clinical value of lncRNA-ATB for diagnostic purpose of liver diseases. Furthermore, BACE1-AS has been examined and tested, particularly in the context of its potential as a therapeutic target for Alzheimer’s disease. Research into BACE1-AS involved understanding its regulatory role on BACE1, an enzyme critical in the formation of amyloid-beta peptides, which are implicated in Alzheimer’s disease pathology [324].

## Conclusion, challenges and future perspectives

Autophagy is a complex biological process that is crucial in maintenance of cellular homeostasis. Aberrations in the autophagy process have been associated with the pathogenesis of various chronic diseases, including cancer, neurodegenerative disorders, metabolic syndrome, and inflammatory conditions. Understanding the mechanisms underlying autophagy and its impact on disease progression provides valuable insights for developing novel therapeutic strategies. LncRNAs exert their regulatory functions through diverse mechanisms, including remodelling of chromatin, transcriptional regulation, post-transcriptional modulation, and protein interaction. In the context of autophagy, lncRNAs have been shown to influence autophagy flux, autophagosome formation, lysosomal function, and autophagy-related signaling pathways. By modulating autophagy-related genes, interacting with autophagy machinery components, and affecting the expression of miRNAs, lncRNAs play diverse roles in autophagy regulation. Perturbations in lncRNA-mediated autophagy regulation have been observed in these diseases, suggesting their potential involvement in disease pathogenesis. For instance, altered expression of lncRNAs such as MALAT1, HOTAIR, and MEG3 has been reported in cancer and linked to disrupted autophagy, contributing to tumor progression or therapy resistance. LncRNAs are hailed as crucial modulators of gene expression, regulating varied biological process of development, differentiation, immunity and homeostasis. Hence, it could not be denied that lncRNAs also play a crucial role in autophagic process in various diseases. In recent years, significant advancements in the field of lncRNAs have led to a growing comprehension of the intricate relationship between disease-associated lncRNAs and autophagy. For example, the role of lncRNAs in cancer appears to be multifaceted, largely due to the dual function of autophagy in tumor progression. Functionally, studies have demonstrated that many lncRNAs regulate autophagy primarily through ceRNA mechanisms, sequestering specific autophagy-related miRNAs. However, lncRNAs also exhibit a more complex involvement in autophagy regulation, influencing processes such as histone and chromatin modifications, transcriptional regulation, and protein–protein interactions, which must be further studied and explored. Our understanding of lncRNA function remains limited due to their low expression levels, poor sequence conservation, and unpredictable roles. However, two key points highlight the need for further investigation of lncRNAs in different regions such as cardiovascular system, lungs, and blood. First, most disease-associated genetic variants are located within noncoding regions, where lncRNAs are prevalent. Second, growing evidence suggests that lncRNAs play significant roles in regulating cellular homeostasis, both in its preservation and disruption. Given the close association between lncRNAs and autophagy, there is potential for the development of lncRNA-based strategies aimed at monitoring or modulating the autophagic flux.

Understanding the functional roles of lncRNAs presents a significant challenge. In contrast to miRNAs, which possess distinct sequence characteristics such as seed sequences and well-defined mechanisms of action including mRNA destabilization or translational inhibition, the functionality of lncRNAs is seldom elucidated by predictable sequence patterns. Moreover, determining the subcellular localization of lncRNAs is crucial for functional analysis. For instance, lncRNAs are known for various interactions, including RNA–RNA, RNA–DNA, and RNA–protein. However, identifying a cytoplasmic localization would prioritize investigations into RNA–RNA or RNA–protein interactions over RNA–DNA interactions [325]. Hence, the primary challenge lies in selecting specific lncRNAs for further investigation from the hundreds identified in an RNA-sequencing analysis. With a moderate sequencing depth of approximately 10 million reads, most lncRNAs exhibits a basal expression level of fewer than 5 fragments per kilobase of transcript per million mapped reads [325]. Initial experiments commonly involve validating the expression levels of a lncRNA through quantitative RT-PCR in the same cells or tissues used in the RNA-sequencing analysis. Subsequent expression studies should encompass a range of cell types (with or without agonists or other stimuli) and tissue types to understand the tissue-specific characteristics of lncRNAs. A significant hurdle in analyzing lncRNA expression is their typically low abundance relative to mRNAs, often resulting in cycle threshold (Ct) values of 35 or higher for most lncRNAs. One possible explanation for the low expression levels of lncRNAs is that it may result from transcriptional noise lacking inherent biological significance [326]. Alternatively, low-abundance lncRNAs could serve functional roles in cis regulatory networks, such as by regulating transcriptional activity either directly or indirectly such as sequestering key transcription factors, scaffolding chromatin remodeling complexes, or facilitating the formation of RNA–DNA triplex structures [327]. Further, the detection and quantification of lncRNAs is also challenging in tissues; the expression levels of most lncRNAs are often too low to be detected through in situ hybridization in tissue sections [325]. Firstly, identifying the localization of lncRNAs is an essential step in understanding their functional roles. Secondly, elucidating the structure of lncRNAs is vital for deepening our comprehension of both their conservation and their functional roles [328]. Recent advancements in techniques, such as RIC-seq and icSHAPE, have provided valuable insights into the secondary and higher-order structures of lncRNAs [329, 330].

Despite their potential, RNA-based therapies comprising lncRNAs encounter significant obstacles. For example, in case of neurological diseases, one major challenge is the inability of most RNA-targeted drugs to cross the blood brain barrier, necessitating intrathecal injection for delivery to the central nervous system, a highly invasive approach that limits their clinical application. Additionally, while lncRNAs are known to perform diverse biological functions with complex mechanisms of action, current research largely focuses on identifying associated miRNAs or binding proteins. Furthermore, lncRNAs exhibit poor conservation across species, adding another layer of complexity to their study. Incorporating knowledge of lncRNAs alongside protein-coding genes and other non-coding genes is essential for elucidating the comprehensive landscape of signaling and transcriptional mechanisms that govern normal homeostatic processes including autophagy. Additionally, this integrated approach is crucial for understanding how these finely regulated systems are disrupted in pathological conditions.

## Data Availability

Not applicable.

## Abbreviations

5-FU: 5-Flurouracil
ADAMTS9-AS1: ADAM metallopeptidase with thrombospondin type 1 motif, 9 (ADAMTS9) antisense RNA 1
AML: Acute myeloid leukemia
AMPK: Adenosine monophosphate-activated protein kinase
ANRIL: Antisense non-coding RNA in the INK4 locus
APF: Autophagy promoting factor
ATB: Activated by transforming growth factor beta
ATG: Autophagy related genes
ATRA: All-trans retinoic acid
BACE1-AS: β-Secretase1 antisense RNA
BANCR: BRAF-activated lncRNA
Bcl-2: B-cell lymphoma 2
BDNS-AS: Brain-derived neurotrophic factor antisense RNA
BLACAT1: Bladder cancer associated transcript 1
CAIF: Cardiac autophagy inhibitory factor
CASC2: Cancer susceptibility candidate 2
CCAT1: Colon cancer-associated transcript-1
ceRNA: Competing endogenous RNAs
Chast: Cardiac hypertrophy-associated transcript
CK-MB: Creatine kinase isoenzyme
CKD: Chronic kidney disease
CMA: Chaperon mediated autophagy
COPD: Chronic obstructive pulmonary disease
CPS1-IT1: CPS1 intronic transcript 1
CRC: Colorectal cancer
CRNDE: Colorectal neoplasia differentially expressed
CUL4A: Cullin 4A
DANCR: Differentiation antagonizing non-protein coding RNA
DANCR: Discrimination antagonizing non-protein coding RNA
DCST1-AS1: DCST1 antisense RNA 1
DFCP1: Double FYVE-containing protein 1
DICER-AS1: DICER1 Antisense RNA 1
EGFR: Epidermal growth factor receptor
EGOT: Eosinophil granule ontogeny transcript
EMT: Epithelial-to-mesenchymal transition
ERS: Endoplasmic reticulum stress
FER1L4: Fer-1 like family member 4
GAS5: Growth arrest-specific transcript 5
GC: Gastric cancer
GSK3: Glycogen synthase kinase-3
H19: H19 imprinted maternally expressed transcript
HAGLROS: HAGLR opposite strand
HIF-1α: Hypoxia-inducible factor 1-alpha
HNF1A-AS1: Hepatocyte nuclear factor 1α antisense RNA 1
HOTAIR: HOX antisense intergenic RNA
HOTAIRM1: HOXA transcript antisense RNA myeloid-specific 1
HULC: Highly upregulated in liver cancer
JPX: JPX transcript, XIST activator
KCNQ1OT1: KCNQ1 opposite strand/antisense transcript 1
LCPAT1: Lung cancer progression-association transcript 1
LDH: Lactate dehydrogenase
Linc-EPS: Long intergenic RNA-erythroid pro-survival
Linc-ROR: LincRNA, regulator of reprogramming
lncRNA: Long non-coding RNA
LVEDD: Left ventricular end diastolic diameter
LVEDP: Left ventricular end diastolic pressure
LVEF: Left ventricular ejection fraction
LVESD: Left ventricular end systolic diameter
LVFS: Left ventricular fractional shortening
MALAT1: Metastasis-associated lung adenocarcinoma transcript 1
MEG3: Maternally expressed gene 3
MHRT: Myosin heavy-chain-associated RNA transcripts
MIAT: Myocardial infarction–associated transcript
miR: MicroRNA
Mst1: Mammalian Ste20-like kinase
mTORC1: Mammalian target of rapamycin complex 1
MTSO2P: Misato homolog 2 pseudogene
NBAT1: Neuroblastoma associated transcript 1
NBR2: Neighbor of BRCA1 gene 2
ncRNA: Non-coding RNA
NEAT1: Nuclear paraspeckle assembly transcript 1
NSCLC: Non-small cell lung cancer
OANCT: Osteoarthritis non-coding transcript
OARSI: Osteoarthritis Research Society International
PANDAR: Promoter of CDKN1A antisense DNA damage activated RNA
PAS: Pre-autophagosomal structure
PI3K: Phosphoinositide 3-kinase
PI3P: Phosphatidylinositol 3-phosphate
Plekhm1: Pleckstrin homology domain-containing protein family M member 1
PPARγ: Peroxisome proliferator-activated receptor γ
PTENP1: Phosphatase and TENsin homolog deleted on chromosome 10 pseudogene-1
PVT1: Plasmacytoma variant translocation 1
RMRP: RNA component of mitochondrial RNA processing endoribonuclease
ROR1-AS1: ROR1 antisense RNA 1
ROS: Reactive oxygen species
SLCO4A1-AS1: SLCO4A1 Antisense RNA 1
SNGH14: Small nucleolar RNA host gene 14
SNHG11: Small nucleolar host gene 11
SNHG6: Small nucleolar RNA host gene 6
SQSTM1: Sequestosome 1
TGFB2-OT1: TGFB2 overlapping transcript 1
TRIM16: Tripartite motif-containing 16
TUG1: Taurine-upregulated gene 1
UCA1: Urothelial carcinoma-associated 1
ULK1: Autophagy activating kinase 1
ZSAF1: Zinc finger nuclear transcription factor, X-box binding 1-type containing 1 antisense RNA 1
